# Supplementary material for: Human Papillomavirus (HPV) Vaccine Coverage and Confidence in Italy: A Nationwide Cross-Sectional Study, the OBVIOUS Project
Source: Vaccines (Basel). 2024 Feb 12;12(2):187. doi: 10.3390/vaccines12020187 (PMC10891781; doi:10.3390/vaccines12020187)
Supplement: Supplementary file 1 [file vaccines-12-00187-s001.zip › vaccines-2852994-supplementary.pdf]

SUPPLEMENTARY TABLES

Table S1. General information about vaccines among participants who answered about their own HPV vaccine uptake, overall and by NUTS.

Characteristic	Italy (*n* = 3160)	Northwestern Italy (*n* = 776)	Northeastern Italy (*n* = 637)	Central Italy (*n* = 634)	Southern Italy (*n* = 736)	Insular Italy (*n* = 377)
Place where you prevalently got vaccines						
Vaccine Hub	2038 (64.5%)	542 (69.8%)	381 (59.8%)	385 (60.7%)	484 (65.8%)	246 (65.3%)
Hospital	653 (20.7%)	135 (17.4%)	175 (27.5%)	140 (22.1%)	130 (17.7%)	73 (19.4%)
Family doctor	310 (9.8%)	63 (8.1%)	43 (6.8%)	81 (12.8%)	81 (11.0%)	42 (11.1%)
Pharmacy	89 (2.8%)	23 (3.0%)	19 (3.0%)	16 (2.5%)	22 (3.0%)	9 (2.4%)
Workplace	36 (1.1%)	7 (0.9%)	9 (1.4%)	9 (1.4%)	8 (1.1%)	3 (0.8%)
Home	34 (1.1%)	6 (0.8%)	10 (1.6%)	3 (0.5%)	11 (1.5%)	4 (1.1%)
Favorite place to get vaccines						
Vaccine Hub	1187 (37.6%)	294 (37.9%)	233 (36.6%)	221 (34.9%)	289 (39.3%)	150 (39.8%)
Hospital	736 (23.3%)	175 (22.6%)	170 (26.7%)	163 (25.7%)	150 (20.4%)	78 (20.7%)
Family doctor	697 (22.1%)	158 (20.4%)	127 (19.9%)	162 (25.6%)	157 (21.3%)	93 (24.7%)
Pharmacy	242 (7.7%)	78 (10.1%)	46 (7.2%)	37 (5.8%)	54 (7.3%)	27 (7.2%)
Home	216 (6.8%)	46 (5.9%)	39 (6.1%)	35 (5.5%)	74 (10.1%)	22 (5.8%)
Workplace	82 (2.6%)	25 (3.2%)	22 (3.5%)	16 (2.5%)	12 (1.6%)	7 (1.9%)
Friends and family’s views on vaccination						
Very unfavorable	121 (3.8%)	30 (3.9%)	35 (5.5%)	17 (2.7%)	27 (3.7%)	12 (3.2%)
Unfavorable	96 (3.0%)	28 (3.6%)	13 (2.0%)	18 (2.8%)	28 (3.8%)	9 (2.4%)
Quite unfavorable	253 (8.0%)	59 (7.6%)	59 (9.3%)	43 (6.8%)	58 (7.9%)	34 (9.0%)
Quite favorable	990 (31.3%)	227 (29.3%)	190 (29.8%)	214 (33.8%)	232 (31.5%)	127 (33.7%)
Favorable	909 (28.8%)	217 (28.0%)	190 (29.8%)	174 (27.4%)	218 (29.6%)	110 (29.2%)
Very favorable	791 (25.0%)	215 (27.7%)	150 (23.5%)	168 (26.5%)	173 (23.5%)	85 (22.5%)

Notes: Piedmont, Aosta Valley, Lombardy, and Liguria constitute Northwestern Italy; Trentino-South Tyrol, Veneto, Friuli-Venezia Giulia, and Emilia-Romagna constitute Northeastern Italy; Tuscany, Umbria, Marche, and Lazio constitute Central Italy; Abruzzo, Molise, Campania, Apulia, Basilicata, and Calabria constitute Southern Italy; Sicily and Sardinia constitute Insular Italy.
HPV, human papillomavirus; *NUTS*, Nomenclature of Territorial Units for Statistics.

Table S2. HPV vaccine uptake in target groups based on age and gender, overall and by NUTS; if the answer is no, the participants are asked whether or not they would get the vaccine.

	All	Yes, I did	No, but I would	No, and I would not
Female participants aged ≥26 y				
Italy	2109 (100.0%)	457 (21.7%)	1203 (57.0%)	449 (21.3%)
Northwestern Italy	551 (100.0%)	109 (19.8%)	324 (58.8%)	118 (21.4%)
Northeastern Italy	419 (100.0%)	115 (27.4%)	214 (51.1%)	90 (21.5%)
Central Italy	429 (100.0%)	79 (18.4%)	258 (60.1%)	92 (21.4%)
Southern Italy	434 (100.0%)	103 (23.7%)	232 (53.5%)	99 (22.8%)
Insular Italy	276 (100.0%)	51 (18.5%)	175 (63.4%)	50 (18.1%)
Male participants aged 18–25 y				
Italy	457 (100.0%)	174 (38.1%)	177 (38.7%)	106 (23.2%)
Northwestern Italy	94 (100.0%)	41 (43.6%)	35 (37.2%)	18 (19.1%)
Northeastern Italy	95 (100.0%)	45 (47.4%)	26 (27.4%)	24 (25.3%)
Central Italy	82 (100.0%)	34 (41.5%)	33 (40.2%)	15 (18.3%)
Southern Italy	143 (100.0%)	40 (28.0%)	70 (49.0%)	33 (23.1%)
Insular Italy	43 (100.0%)	14 (32.6%)	13 (30.2%)	16 (37.2%)
Female participants aged 18–25 y				
Italy	594 (100.0%)	480 (80.8%)	81 (13.6%)	33 (5.6%)
Northwestern Italy	131 (100.0%)	109 (83.2%)	15 (11.5%)	7 (5.3%)
Northeastern Italy	123 (100.0%)	105 (85.4%)	10 (8.1%)	8 (6.5%)
Central Italy	123 (100.0%)	99 (80.5%)	18 (14.6%)	6 (4.9%)
Southern Italy	159 (100.0%)	124 (78.0%)	27 (17.0%)	8 (5.0%)
Insular Italy	58 (100.0%)	43 (74.1%)	11 (19.0%)	4 (6.9%)
Male offspring aged 9–11 y				
Italy	226 (100.0%)	91 (40.3%)	96 (42.5%)	39 (17.3%)
Northwestern Italy	61 (100.0%)	28 (45.9%)	22 (36.1%)	11 (18.0%)
Northeastern Italy	47 (100.0%)	20 (42.6%)	17 (36.2%)	10 (21.3%)
Central Italy	47 (100.0%)	14 (29.8%)	25 (53.2%)	8 (17.0%)
Southern Italy	50 (100.0%)	15 (30.0%)	26 (52.0%)	9 (18.0%)
Insular Italy	21 (100.0%)	14 (66.7%)	6 (28.6%)	1 (4.8%)
Female offspring aged 9–11 y				
Italy	201 (100.0%)	80 (39.8%)	100 (49.8%)	21 (10.4%)
Northwestern Italy	58 (100.0%)	26 (44.8%)	26 (44.8%)	6 (10.3%)
Northeastern Italy	28 (100.0%)	13 (46.4%)	12 (42.9%)	3 (10.7%)
Central Italy	35 (100.0%)	12 (34.3%)	19 (54.3%)	4 (11.4%)
Southern Italy	56 (100.0%)	21 (37.5%)	31 (55.4%)	4 (7.1%)
Insular Italy	24 (100.0%)	8 (33.3%)	12 (50.0%)	4 (16.7%)
Male offspring aged 12–17 y				
Italy	431 (100.0%)	220 (51.0%)	131 (30.4%)	80 (18.6%)
Northwestern Italy	137 (100.0%)	69 (50.4%)	37 (27.0%)	31 (22.6%)
Northeastern Italy	71 (100.0%)	46 (64.8%)	14 (19.7%)	11 (15.5%)
Central Italy	79 (100.0%)	41 (51.9%)	25 (31.6%)	13 (16.5%)
Southern Italy	97 (100.0%)	45 (46.4%)	33 (34.0%)	19 (19.6%)
Insular Italy	47 (100.0%)	19 (40.4%)	22 (46.8%)	6 (12.8%)
Female offspring aged 12–17 y				
Italy	408 (100.0%)	299 (73.3%)	69 (16.9%)	40 (9.8%)
Northwestern Italy	127 (100.0%)	99 (78.0%)	13 (10.2%)	15 (11.8%)
Northeastern Italy	61 (100.0%)	43 (70.5%)	11 (18.0%)	7 (11.5%)
Central Italy	71 (100.0%)	48 (67.6%)	16 (22.5%)	7 (9.9%)
Southern Italy	93 (100.0%)	73 (78.5%)	14 (15.1%)	6 (6.5%)

Insular Italy	56 (100.0%)	36 (64.3%)	15 (26.8%)	5 (8.9%)

Notes: Females include non-binary people and participants who did not disclose their gender identity. Information about offspring was provided by the parents of the children. Piedmont, Aosta Valley, Lombardy, and Liguria constitute Northwestern Italy; Trentino-South Tyrol, Veneto, Friuli-Venezia Giulia, and Emilia-Romagna constitute Northeastern Italy; Tuscany, Umbria, Marche, and Lazio constitute Central Italy; Abruzzo, Molise, Campania, Apulia, Basilicata, and Calabria constitute Southern Italy; Sicily and Sardinia constitute Insular Italy.
HPV, human papillomavirus; *NUTS*, Nomenclature of Territorial Units for Statistics.

Table S3. Information about HPV vaccination among participants who answered about their own HPV vaccine uptake, by target group based on age and gender.

Characteristic	Male participants aged 18–25 y (*n* = 457)	Female participants aged 18–25 y (*n* = 594)	Female participants aged ≥26 y (*n* = 2109)
Worry about getting HPV infection			
Not worried	142 (31.1%)	106 (17.8%)	349 (16.5%)
A little worried	224 (49.0%)	293 (49.3%)	933 (44.2%)
Quite worried	80 (17.5%)	165 (27.8%)	639 (30.3%)
Very worried	11 (2.4%)	30 (5.1%)	188 (8.9%)
Perception of the safety of HPV vaccine			
Very safe	143 (31.3%)	204 (34.3%)	506 (24.0%)
Quite safe	265 (58.0%)	348 (58.6%)	1291 (61.2%)
Quite unsafe	44 (9.6%)	35 (5.9%)	254 (12.0%)
Very unsafe	5 (1.1%)	7 (1.2%)	58 (2.8%)
Belief about having higher priority for HPV vaccination			
Yes	146 (31.9%)	435 (73.2%)	587 (27.8%)
No	108 (23.6%)	38 (6.4%)	624 (29.6%)
Don’t know	203 (44.4%)	121 (20.4%)	898 (42.6%)
Perception of how easy it is to access healthcare facilities to get an HPV vaccine			
Very easy	66 (14.4%)	161 (27.1%)	266 (12.6%)
Quite easy	278 (60.8%)	344 (57.9%)	1217 (57.7%)
Quite difficult	101 (22.1%)	74 (12.5%)	509 (24.1%)
Very difficult	12 (2.6%)	15 (2.5%)	117 (5.5%)
Perception of the affordability of HPV vaccines			
Very affordable	108 (23.6%)	143 (24.1%)	311 (14.7%)
Quite affordable	261 (57.1%)	346 (58.2%)	1120 (53.1%)
Little affordable	83 (18.2%)	89 (15.0%)	554 (26.3%)
Not at all affordable	5 (1.1%)	16 (2.7%)	124 (5.9%)
Payment for HPV vaccination*			
Gratis	100 (57.5%)	363 (75.6%)	295 (64.6%)
For a fee	36 (20.7%)	33 (6.9%)	97 (21.2%)
Don’t remember	38 (21.8%)	84 (17.5%)	65 (14.2%)

*Restricted to subjects vaccinated against HPV.
HPV, human papillomavirus.

Table S4. Information about HPV vaccination among participants who answered about their youngest children's HPV vaccine uptake, by target group based on age and gender.

Characteristic	Male offspring aged 9–11 y (*n* = 226)	Female offspring aged 9–11 y (*n* = 201)	Male offspring aged 12–17 y (*n* = 431)	Female offspring aged 12–17 y (*n* = 408)
Worry about getting HPV infection				
Not worried	30 (13.3%)	20 (10.0%)	74 (17.2%)	54 (13.2%)
A little worried	91 (40.3%)	75 (37.3%)	200 (46.4%)	161 (39.5%)
Quite worried	89 (39.4%)	87 (43.3%)	133 (30.9%)	150 (36.8%)
Very worried	16 (7.1%)	19 (9.5%)	24 (5.6%)	43 (10.5%)
Perception of the safety of HPV vaccine				
Very safe	48 (21.2%)	57 (28.4%)	106 (24.6%)	96 (23.5%)
Quite safe	139 (61.5%)	120 (59.7%)	253 (58.7%)	265 (65.0%)
Quite unsafe	28 (12.4%)	15 (7.5%)	52 (12.1%)	33 (8.1%)
Very unsafe	11 (4.9%)	9 (4.5%)	20 (4.6%)	14 (3.4%)
Belief about having higher priority for HPV vaccination				
Yes	116 (51.3%)	131 (65.2%)	246 (57.1%)	327 (80.1%)
No	44 (19.5%)	22 (10.9%)	60 (13.9%)	22 (5.4%)
Don't know	66 (29.2%)	48 (23.9%)	125 (29.0%)	59 (14.5%)
Perception of how easy it is to access healthcare facilities to get an HPV vaccine				
Very easy	56 (24.8%)	52 (25.9%)	94 (21.8%)	114 (27.9%)
Quite easy	122 (54.0%)	123 (61.2%)	266 (61.7%)	236 (57.8%)
Quite difficult	39 (17.3%)	19 (9.5%)	47 (10.9%)	44 (10.8%)
Very difficult	9 (4.0%)	7 (3.5%)	24 (5.6%)	14 (3.4%)
Perception of the affordability of HPV vaccines				
Very affordable	73 (32.3%)	79 (39.3%)	128 (29.7%)	142 (34.8%)
Quite affordable	116 (51.3%)	99 (49.3%)	229 (53.1%)	213 (52.2%)
Little affordable	31 (13.7%)	21 (10.4%)	59 (13.7%)	43 (10.5%)
Not at all affordable	6 (2.7%)	2 (1.0%)	15 (3.5%)	10 (2.5%)
Payment for HPV vaccination*				
Gratis	74 (81.3%)	67 (83.8%)	179 (81.4%)	266 (89.0%)
For a fee	13 (14.3%)	6 (7.5%)	28 (12.7%)	20 (6.7%)
Don't remember	4 (4.4%)	7 (8.8%)	13 (5.9%)	13 (4.3%)

*Restricted to subjects vaccinated against HPV.
HPV, human papillomavirus

Table S5. Predictors of HPV vaccine uptake and hesitancy (expressed as delay vs. refusal) among participants who answered on their own behalf (*n* = 3160).

Characteristic	Did get the vaccine			Would get the vaccine			Would not get the vaccine		
	Predicted probability	Discrete difference (*Δ*)		Predicted probability	Discrete difference (*Δ*)		Predicted probability	Discrete difference (*Δ*)	
		Estimate	95% CI		Estimate	95% CI		Estimate	95% CI
Gender									
Male	21.1%	Ref.		54.6%	Ref.		24.2%	Ref.	
Female†	39.5%	18.3*	14.5, 22.2	43.6%	−11.0*	−18.1, −3.9	16.9%	−7.3*	−13.6, −1.1
Age group, y									
18–25	62.9%	Ref.		24.2%	Ref.		13.0%	Ref.	
26–32	35.1%	−27.8*	−34.5, −21.1	46.8%	22.7*	15.6, 29.7	18.1%	5.1*	0.0, 10.3
33–44	21.9%	−41.0*	−47.0, −35.0	59.7%	35.5*	29.1, 41.9	18.4%	5.5*	0.9, 10.0
45–59	13.3%	−49.6*	−55.6, −43.6	60.3%	36.2*	29.3, 43.1	26.4%	13.4*	8.1, 18.8
NUTS statistical region									
Northwestern Italy	34.9%	Ref.		46.3%	Ref.		18.8%	Ref.	
Northeastern Italy	38.7%	3.8	−0.3, 7.8	43.1%	−3.2	−7.7, 1.3	18.2%	−0.6	−3.9, 2.8
Central Italy	32.6%	−2.3	−6.2, 1.6	48.8%	2.5	−2.0, 6.9	18.6%	−0.2	−3.5, 3.2
Southern Italy	35.7%	0.8	−3.1, 4.6	44.8%	−1.5	−5.9, 2.8	19.5%	0.7	−2.5, 4.0
Insular Italy	32.8%	−2.2	−6.8, 2.5	49.7%	3.4	−1.8, 8.7	17.5%	−1.3	−5.1, 2.5
Degree of urbanization‡									
City	34.0%	Ref.		47.1%	Ref.		18.9%	Ref.	
Town or suburb	35.9%	1.9	−1.0, 4.8	46.0%	−1.1	−4.4, 2.1	18.1%	−0.7	−3.2, 1.7
Rural area	35.6%	1.5	−2.6, 5.7	45.0%	−2.1	−6.8, 2.5	19.5%	0.6	−2.9, 4.1
Educational attainment									
Academic/Post-graduate degree	35.2%	Ref.		46.1%	Ref.		18.7%	Ref.	
High school diploma	35.6%	0.4	−2.5, 3.4	45.9%	−0.2	−3.6, 3.2	18.4%	−0.3	−2.9, 2.4
Less than high school diploma	31.8%	−3.4	−8.4, 1.5	48.9%	2.9	−2.7, 8.5	19.3%	0.6	−3.4, 4.6
Worry about HPV infection									
Very worried	35.2%	Ref.		52.9%	Ref.		11.9%	Ref.	
Quite worried	36.3%	1.0	−4.5, 6.6	51.3%	−1.6	−8.0, 4.8	12.4%	0.5	−4.2, 5.3
A little worried	34.4%	−0.9	−6.3, 4.5	48.4%	−4.5	−10.7, 1.8	17.2%	5.3*	0.7, 10.0
Not worried	36.9%	1.7	−4.3, 7.6	31.1%	−21.8*	−28.7, −14.9	31.9%	20.1*	14.6, 25.6
Perception of vaccine safety									
Very safe	40.6%	Ref.		50.2%	Ref.		9.1%	Ref.	

Quite safe	34.6%	−6.0*	−9.6, −2.5	50.9%	0.6	−3.3, 4.6	14.6%	5.4*	2.7, 8.1
Quite/Very unsafe	27.4%	−13.2*	−18.4, −7.9	21.9%	−28.3*	−33.9, −22.7	50.6%	41.5*	36.1, 47.0
Dear ones' views on vaccination in general									
Very favorable	34.2%	Ref.		52.5%	Ref.		13.3%	Ref.	
Favorable	36.5%	2.4	−1.2, 6.0	47.6%	−5.0*	−9.2, −0.8	15.9%	2.6	−0.5, 5.7
Quite favorable	35.1%	1.0	−2.7, 4.7	44.9%	−7.6*	−11.9, −3.3	19.9%	6.6*	3.4, 9.8
Quite unfavorable	36.4%	2.2	−3.5, 8.0	35.6%	−16.9*	−23.3, −10.5	28.0%	14.7*	9.5, 19.9
Unfavorable/Very unfavorable	34.8%	0.7	−5.5, 6.9	40.1%	−12.5*	−19.7, −5.3	25.1%	11.8*	6.2, 17.3
Belief about having higher priority for vaccination§									
Yes (right)	53.8%	Ref.		33.7%	Ref.		12.5%	Ref.	
Yes (wrong)	53.2%	−0.6	−9.2, 8.0	37.9%	4.2	−4.8, 13.1	8.9%	−3.6	−9.4, 2.3
No	19.9%	−33.9*	−42.8, −25.0	53.7%	20.0*	10.6, 29.4	26.4%	13.9*	7.5, 20.4
Don't know	26.1%	−27.7*	−36.1, −19.3	53.0%	19.3*	10.4, 28.1	20.9%	8.4*	2.4, 14.4
Perceived ease of access to get the vaccine									
Very easy	38.0%	Ref.		39.5%	Ref.		22.5%	Ref.	
Quite easy	34.6%	−3.5	−7.7, 0.7	45.6%	6.1*	1.1, 11.1	19.8%	−2.6	−7.0, 1.7
Quite/Very difficult	34.2%	−3.8	−8.8, 1.1	50.4%	10.9*	5.1, 16.7	15.4%	−7.0*	−11.7, −2.4
Perceived affordability of the vaccine									
Very affordable	38.5%	Ref.		43.1%	Ref.		18.5%	Ref.	
Quite affordable	34.5%	−3.9	−8.0, 0.1	46.3%	3.2	−1.4, 7.8	19.2%	0.8	−2.8, 4.3
Little/Not at all affordable	34.1%	−4.4	−9.0, 0.3	48.2%	5.1	−0.1, 10.4	17.7%	−0.8	−4.7, 3.2

*P-value ≤0.05, i.e., Δ significantly ≠ 0.
†Including non-binary people and participants who did not disclose their gender identity.
‡Graded using the Eurostat Degree of Urbanization (DEGURBA) classification system.
§Those correctly aware of having higher priority for vaccination are female participants born between 1997 and 2004 or male participants born between 2003 and 2004.
HPV, human papillomavirus; *NUTS*, Nomenclature of Territorial Units for Statistics.

Table S6. Predictors of HPV vaccine uptake and hesitancy (expressed as delay vs. refusal) among participants who answered on their children's behalf (n = 1266).

Characteristic	Did get the vaccine			Would get the vaccine			Would not get the vaccine		
	Predicted probability	Discrete difference (*Δ*)		Predicted probability	Discrete difference (*Δ*)		Predicted probability	Discrete difference (*Δ*)	
		Estimate	95% CI		Estimate	95% CI		Estimate	95% CI
Parent's gender									
Male	60.0%	Ref.		26.2%	Ref.		13.9%	Ref.	
Female†	50.0%	−10.0*	−14.8, −5.2	35.6%	9.4*	4.6, 14.2	14.4%	0.6	−2.2, 3.3
Parent's age group, y									
18–34	60.5%	Ref.		26.8%	Ref.		12.7%	Ref.	
35–44	53.6%	−6.9	−17.5, 3.8	31.5%	4.7	−5.4, 14.8	14.9%	2.2	−4.2, 8.5
45–54	54.9%	−5.6	−15.9, 4.8	31.0%	4.2	−5.6, 14.1	14.0%	1.3	−4.8, 7.4
≥55	52.8%	−7.7	−19.0, 3.5	33.0%	6.2	−4.6, 17.0	14.2%	1.5	−5.0, 8.0
NUTS statistical region									
Northwestern Italy	57.4%	Ref.		27.7%	Ref.		15.0%	Ref.	
Northeastern Italy	59.6%	2.2	−4.8, 9.2	25.4%	−2.3	−9.1, 4.5	15.0%	0.1	−4.2, 4.3
Central Italy	49.9%	−7.4*	−14.3, −0.6	34.5%	6.8	0.0, 13.7	15.6%	0.6	−3.6, 4.8
Southern Italy	52.0%	−5.3	−11.8, 1.2	35.8%	8.1*	1.6, 14.6	12.2%	−2.8	−6.4, 0.9
Insular Italy	52.2%	−5.1	−13.1, 2.8	34.7%	7.1	−0.9, 15.0	13.0%	−1.9	−6.4, 2.5
Degree of urbanization‡									
City	55.3%	Ref.		30.8%	Ref.		13.9%	Ref.	
Town or suburb	54.2%	−1.1	−6.2, 3.9	31.7%	0.9	−4.1, 5.9	14.1%	0.2	−2.7, 3.1
Rural area	52.7%	−2.6	−10.6, 5.5	31.5%	0.7	−7.2, 8.6	15.8%	1.9	−3.1, 6.9
Parent's educational attainment									
Academic/Post-graduate degree	56.8%	Ref.		31.1%	Ref.		12.1%	Ref.	
High school diploma	54.6%	−2.2	−7.6, 3.3	31.0%	−0.1	−5.5, 5.3	14.4%	2.2	−0.8, 5.3
Less than high school diploma	47.6%	−9.2*	−17.8, −0.7	33.9%	2.8	−5.8, 11.5	18.6%	6.4*	1.1, 11.7
Child's gender									
Male	51.1%	Ref.		34.2%	Ref.		14.7%	Ref.	
Female	58.1%	7.0*	2.2, 11.9	28.3%	−6.0*	−10.8, −1.2	13.6%	−1.1	−3.9, 1.8
Child's age group, y									
9–11	46.1%	Ref.		40.4%	Ref.		13.5%	Ref.	
12–17	59.2%	13.1*	5.9, 20.4	26.1%	−14.3*	−21.4, −7.2	14.7%	1.2	−2.2, 4.6
Worry about HPV infection									

Very worried	59.3%	Ref.		30.9%	Ref.		9.8%	Ref.	
Quite worried	53.4%	−5.9	−15.3, 3.5	36.3%	5.4	−4.3, 15.1	10.3%	0.4	−7.1, 7.9
A little worried	54.8%	−4.5	−14.0, 5.0	32.2%	1.3	−8.4, 11.1	13.0%	3.1	−4.2, 10.5
Not worried	58.0%	−1.3	−12.2, 9.7	15.5%	−15.3*	−25.9, −4.8	26.5%	16.6*	7.7, 25.5
Perception of vaccine safety for the child									
Very safe	62.3%	Ref.		35.2%	Ref.		2.5%	Ref.	
Quite safe	56.9%	−5.4	−12.2, 1.3	34.1%	−1.1	−7.9, 5.6	9.1%	6.6*	3.7, 9.5
Quite/Very unsafe	34.9%	−27.4*	−38.9, −15.9	19.9%	−15.3*	−25.3, −5.4	45.2%	42.7*	32.6, 52.9
Dear ones’ views on vaccination in general									
Very favorable	52.2%	Ref.		34.4%	Ref.		13.4%	Ref.	
Favorable	51.9%	−0.3	−7.2, 6.7	34.1%	−0.3	−7.4, 6.7	14.0%	0.6	−4.4, 5.6
Quite favorable	58.2%	6.0	−1.0, 13.0	28.3%	−6.1	−13.2, 0.9	13.5%	0.1	−4.7, 4.9
Quite unfavorable	56.2%	4.0	−6.7, 14.7	31.5%	−2.9	−13.7, 8.0	12.3%	−1.1	−6.9, 4.7
Unfavorable/Very unfavorable	57.0%	4.8	−7.6, 17.2	23.5%	−10.9	−23.1, 1.4	19.5%	6.1	−1.2, 13.4
Belief that the child has priority for vaccination§									
Yes (right)	70.3%	Ref.		21.9%	Ref.		7.9%	Ref.	
Yes (wrong)	62.3%	−8.0	−18.0, 2.1	30.7%	8.8	−0.3, 17.9	7.0%	−0.9	−5.7, 4.0
No	24.7%	−45.6*	−55.0, −36.2	46.4%	24.5*	14.5, 34.5	28.9%	21.1*	13.6, 28.5
Don’t know	31.7%	−38.6*	−45.7, −31.5	49.2%	27.3*	20.4, 34.3	19.1%	11.3*	6.9, 15.6
Perceived ease of access for the child to get the vaccine									
Very easy	60.4%	Ref.		26.6%	Ref.		13.0%	Ref.	
Quite easy	51.4%	−8.9*	−15.4, −2.5	32.6%	5.9	−0.4, 12.3	16.0%	3.0	−1.1, 7.1
Quite/Very difficult	55.6%	−4.7	−14.0, 4.5	33.4%	6.8	−2.3, 15.9	10.9%	−2.1	−6.8, 2.6
Perceived affordability of the vaccine for the child									
Very affordable	51.3%	Ref.		33.5%	Ref.		15.2%	Ref.	
Quite affordable	55.1%	3.8	−2.2, 9.8	30.7%	−2.8	−9.0, 3.4	14.3%	−1.0	−4.9, 3.0
Little/Not at all affordable	59.8%	8.5*	0.0, 17.0	27.2%	−6.3	−14.8, 2.2	13.0%	−2.2	−7.0, 2.7

**P*-value ≤0.05, i.e., *Δ* significantly ≠ 0.

†Including non-binary people and participants who did not disclose their gender identity.

‡Graded using the Eurostat Degree of Urbanization (DEGURBA) classification system.

§Those correctly aware that their children have higher priority for vaccination are parents of offspring born between 2003 and 2010.

HPV, human papillomavirus; *NUTS*, Nomenclature of Territorial Units for Statistics.

SUPPLEMENTARY FIGURES

Figure S1. Worry about getting HPV infection among female participants 26 years of age and older who answered on their own behalf (*n* = 2109), overall and by NUTS.

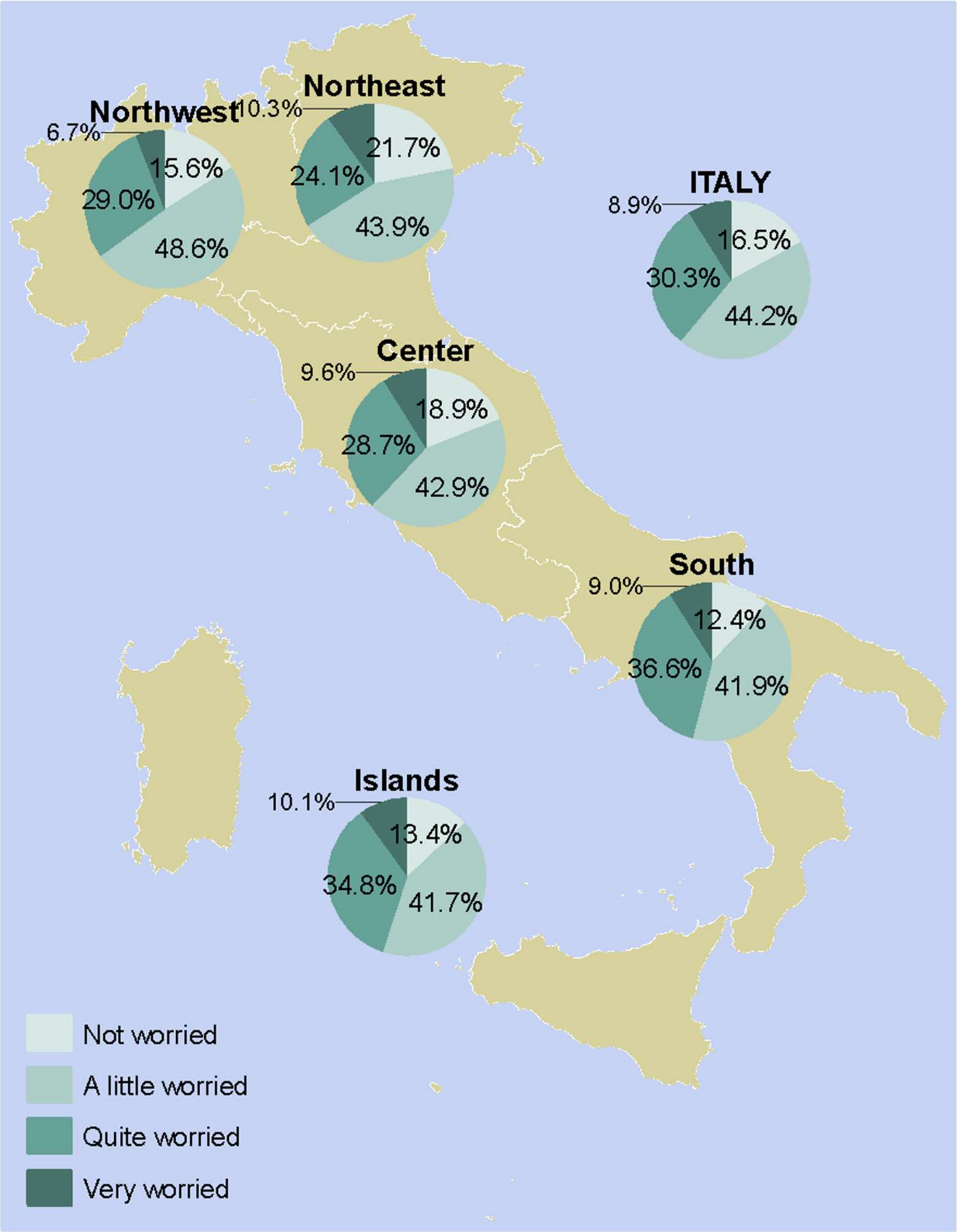

Notes: Females include non-binary people and participants who did not disclose their gender identity. Piedmont, Aosta Valley, Lombardy, and Liguria constitute Northwestern Italy; Trentino-South Tyrol, Veneto, Friuli-Venezia Giulia, and Emilia-Romagna constitute Northeastern Italy; Tuscany, Umbria, Marche, and Lazio constitute Central Italy; Abruzzo, Molise, Campania, Apulia, Basilicata, and Calabria constitute Southern Italy; Sicily and Sardinia constitute Insular Italy.
HPV, human papillomavirus; *NUTS*, Nomenclature of Territorial Units for Statistics.

Figure S2. Worry about getting HPV infection among male vs. female participants between 18 and 25 years of age who answered on their own behalf (n = 1051), overall and by NUTS (%).

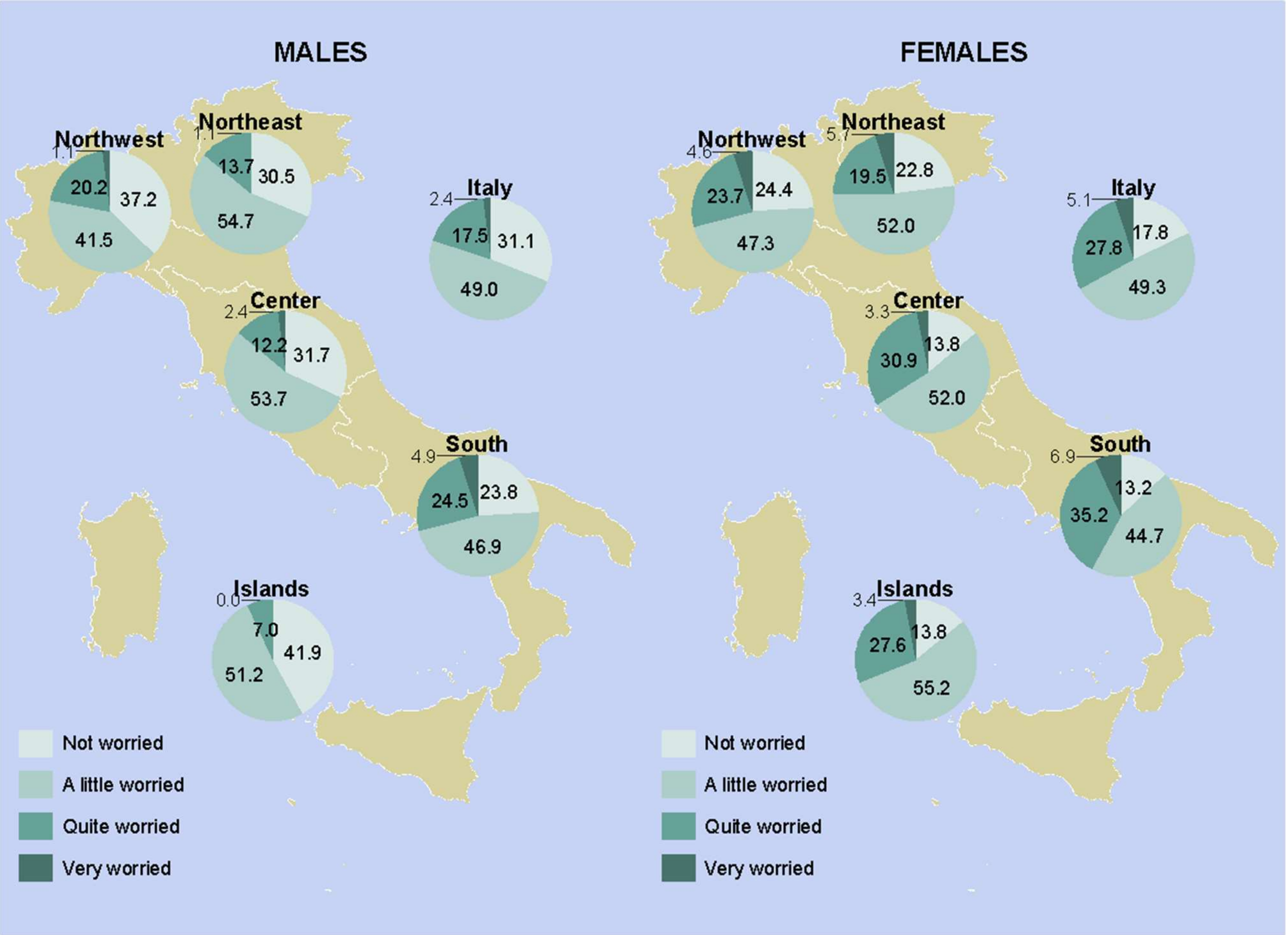

Notes: Females include non-binary people and participants who did not disclose their gender identity. Piedmont, Aosta Valley, Lombardy, and Liguria constitute Northwestern Italy; Trentino-South Tyrol, Veneto, Friuli-Venezia Giulia, and Emilia-Romagna constitute Northeastern Italy; Tuscany, Umbria, Marche, and Lazio constitute Central Italy; Abruzzo, Molise, Campania, Apulia, Basilicata, and Calabria constitute Southern Italy; Sicily and Sardinia constitute Insular Italy.
HPV, human papillomavirus; *NUTS*, Nomenclature of Territorial Units for Statistics.

Figure S3. Worry about getting HPV infection among male vs. female offspring between 9 and 11 years of age (n = 427), overall and by NUTS (%).

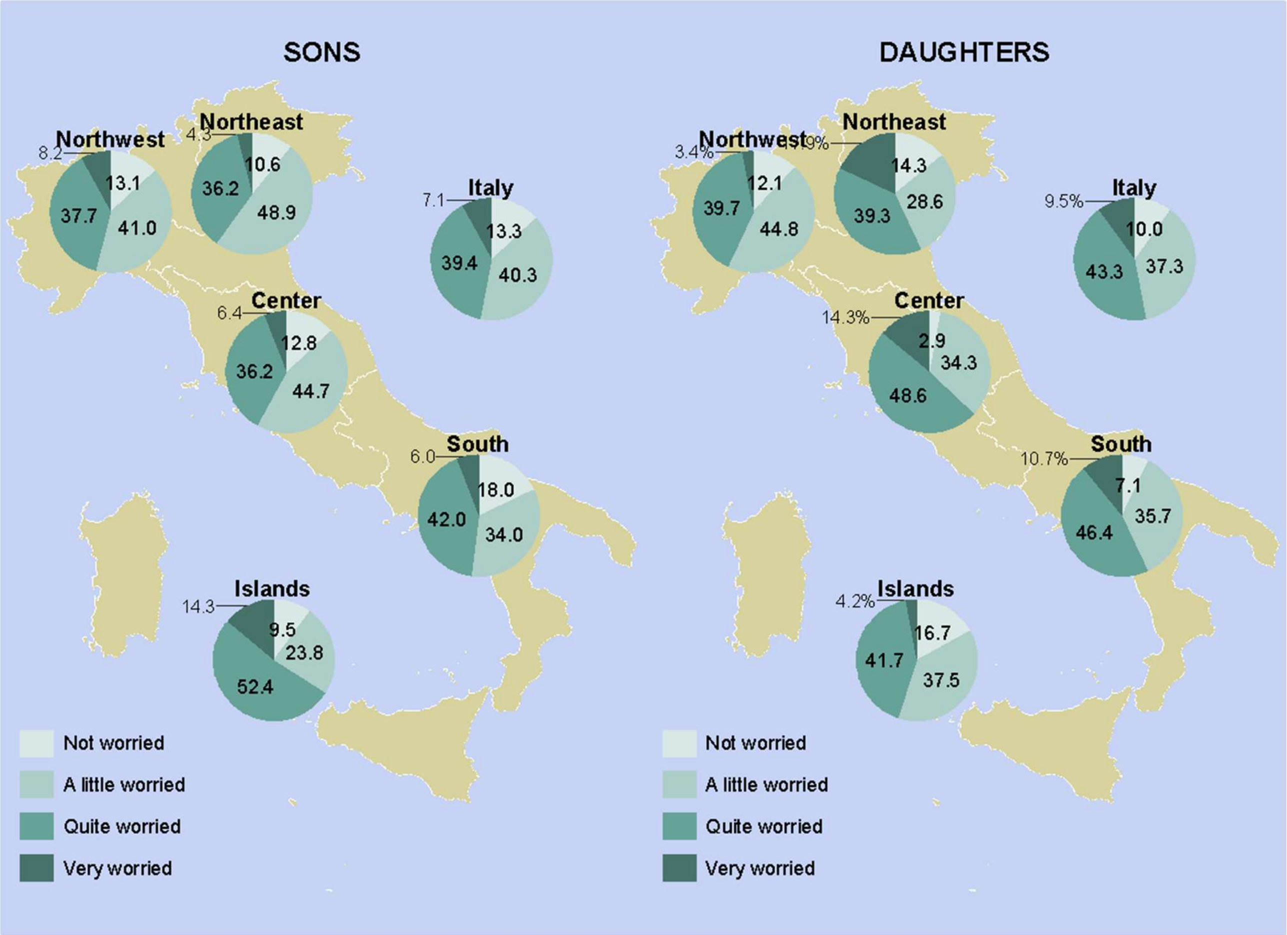

Notes: Information was provided by the parents of the children. Piedmont, Aosta Valley, Lombardy, and Liguria constitute Northwestern Italy; Trentino-South Tyrol, Veneto, Friuli-Venezia Giulia, and Emilia-Romagna constitute Northeastern Italy; Tuscany, Umbria, Marche, and Lazio constitute Central Italy; Abruzzo, Molise, Campania, Apulia, Basilicata, and Calabria constitute Southern Italy; Sicily and Sardinia constitute Insular Italy.
HPV, human papillomavirus; *NUTS*, Nomenclature of Territorial Units for Statistics.

Figure S4. Worry about getting HPV infection among male vs. female offspring between 12 and 17 years of age (*n* = 839), overall and by NUTS (%).

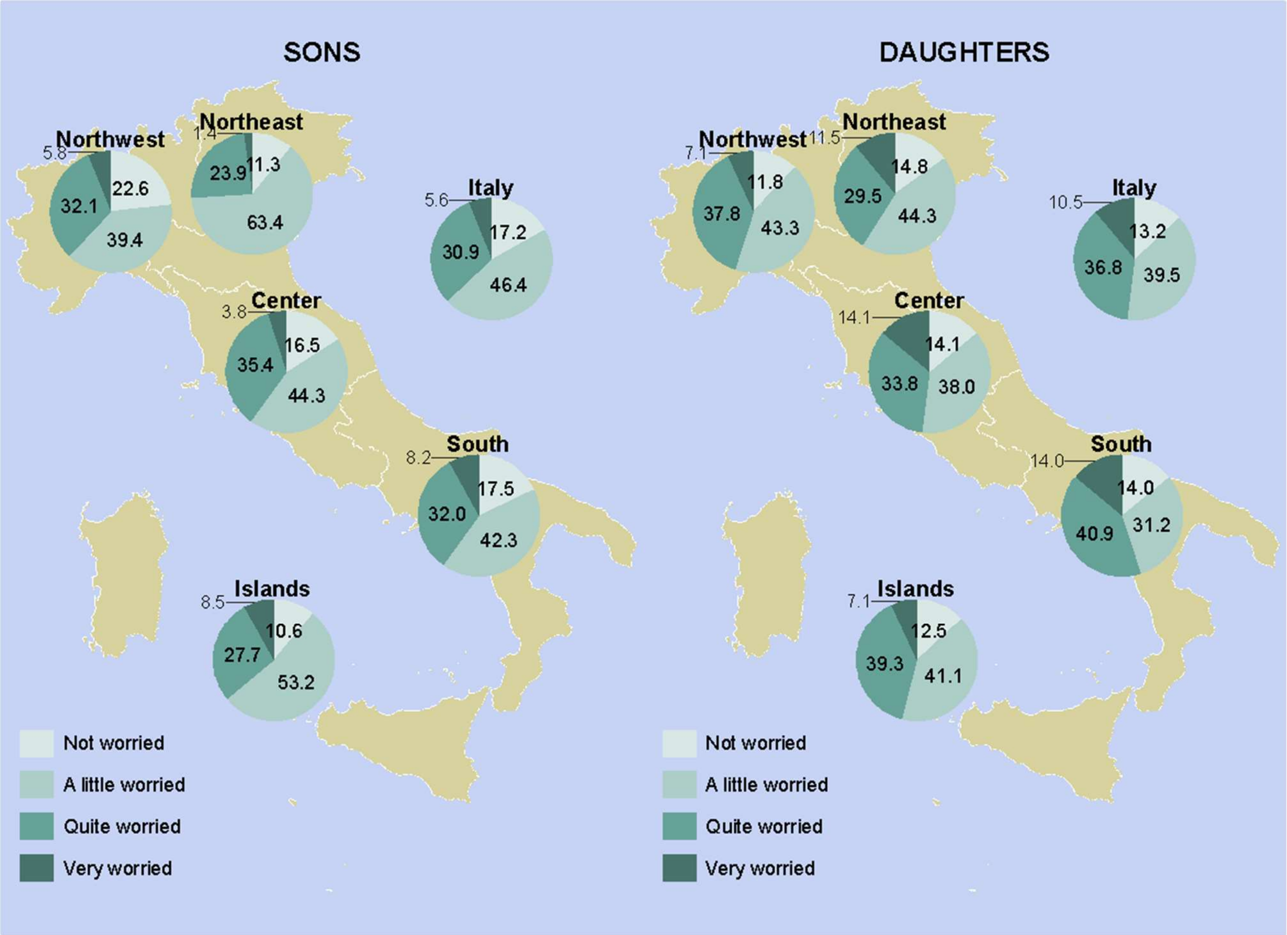

Notes: Information was provided by the parents of the children. Piedmont, Aosta Valley, Lombardy, and Liguria constitute Northwestern Italy; Trentino-South Tyrol, Veneto, Friuli-Venezia Giulia, and Emilia-Romagna constitute Northeastern Italy; Tuscany, Umbria, Marche, and Lazio constitute Central Italy; Abruzzo, Molise, Campania, Apulia, Basilicata, and Calabria constitute Southern Italy; Sicily and Sardinia constitute Insular Italy.
HPV, human papillomavirus; *NUTS*, Nomenclature of Territorial Units for Statistics.

Figure S5. Perception of the safety of HPV vaccine among female participants 26 years of age and older who answered on their own behalf (n = 2109), overall and by NUTS.

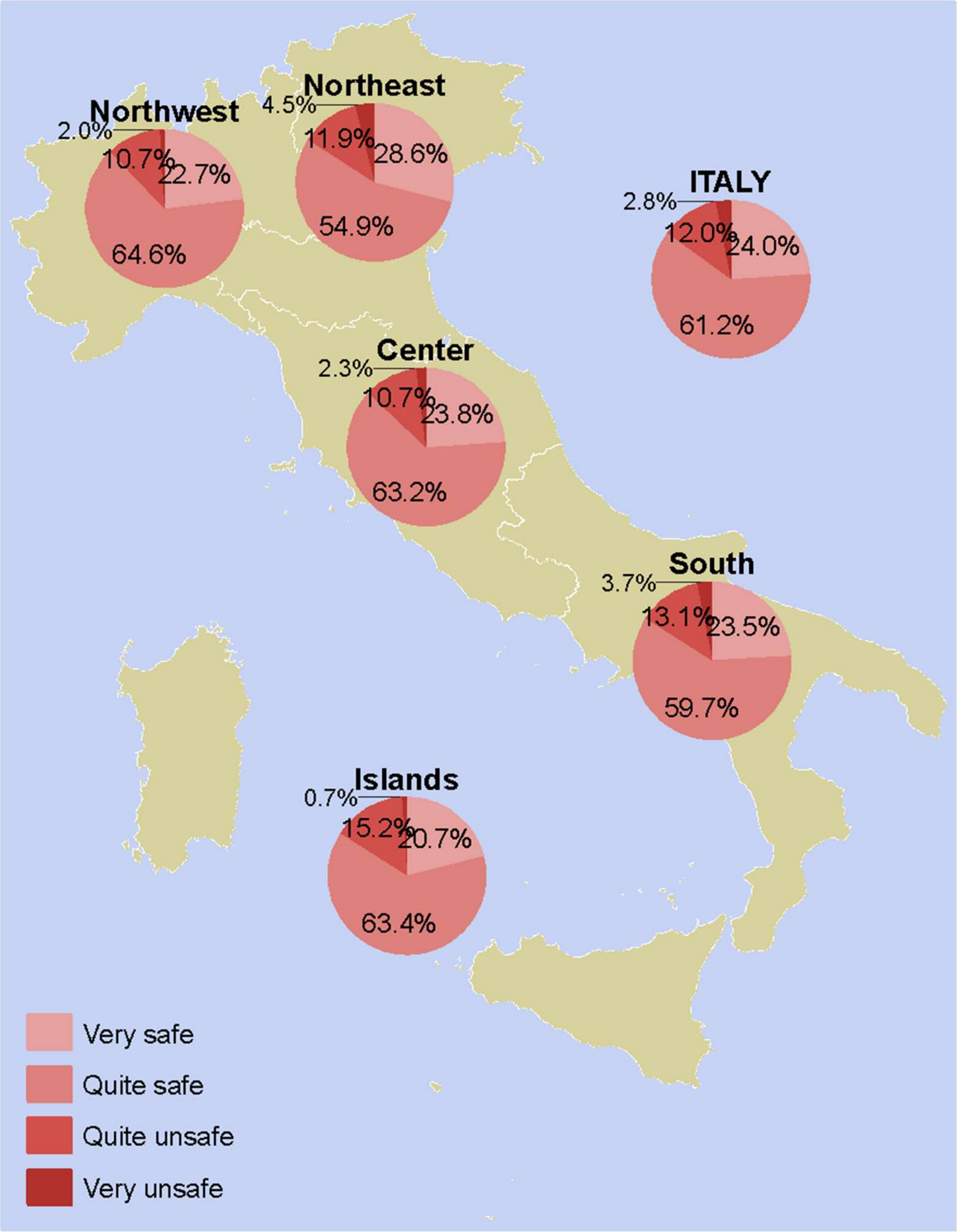

Notes: Females include non-binary people and participants who did not disclose their gender identity. Piedmont, Aosta Valley, Lombardy, and Liguria constitute Northwestern Italy; Trentino-South Tyrol, Veneto, Friuli-Venezia Giulia, and Emilia-Romagna constitute Northeastern Italy; Tuscany, Umbria, Marche, and Lazio constitute Central Italy; Abruzzo, Molise, Campania, Apulia, Basilicata, and Calabria constitute Southern Italy; Sicily and Sardinia constitute Insular Italy.
HPV, human papillomavirus; *NUTS*, Nomenclature of Territorial Units for Statistics.

Figure S6. Perception of the safety of HPV vaccine among male vs. female participants between 18 and 25 years of age who answered on their own behalf (n = 1051), overall and by NUTS.

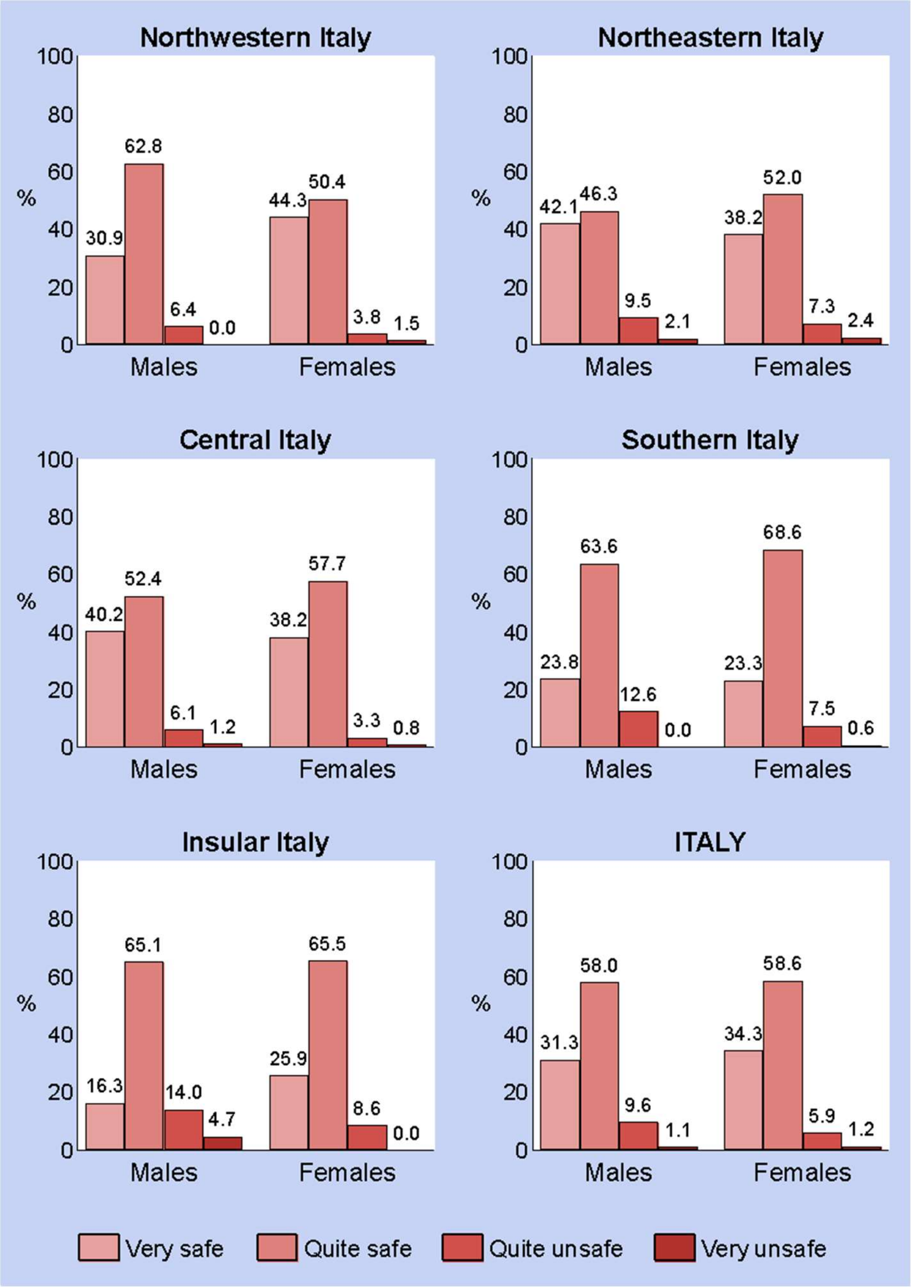

Notes: The presence of zero counts made it impossible to visualize the data as thematic maps using the 'spmap' Stata command. Females include non-binary people and participants who did not disclose their gender identity. Piedmont, Aosta Valley, Lombardy, and Liguria constitute Northwestern Italy; Trentino-South Tyrol, Veneto, Friuli-Venezia Giulia, and Emilia-Romagna constitute Northeastern Italy; Tuscany, Umbria, Marche, and Lazio constitute Central Italy; Abruzzo, Molise, Campania, Apulia, Basilicata, and Calabria constitute Southern Italy; Sicily and Sardinia constitute Insular Italy.

HPV, human papillomavirus; *NUTS*, Nomenclature of Territorial Units for Statistics.

Figure S7. Perception of the safety of HPV vaccine among male vs. female offspring between 9 and 11 years of age (*n* = 427), overall and by NUTS (%).

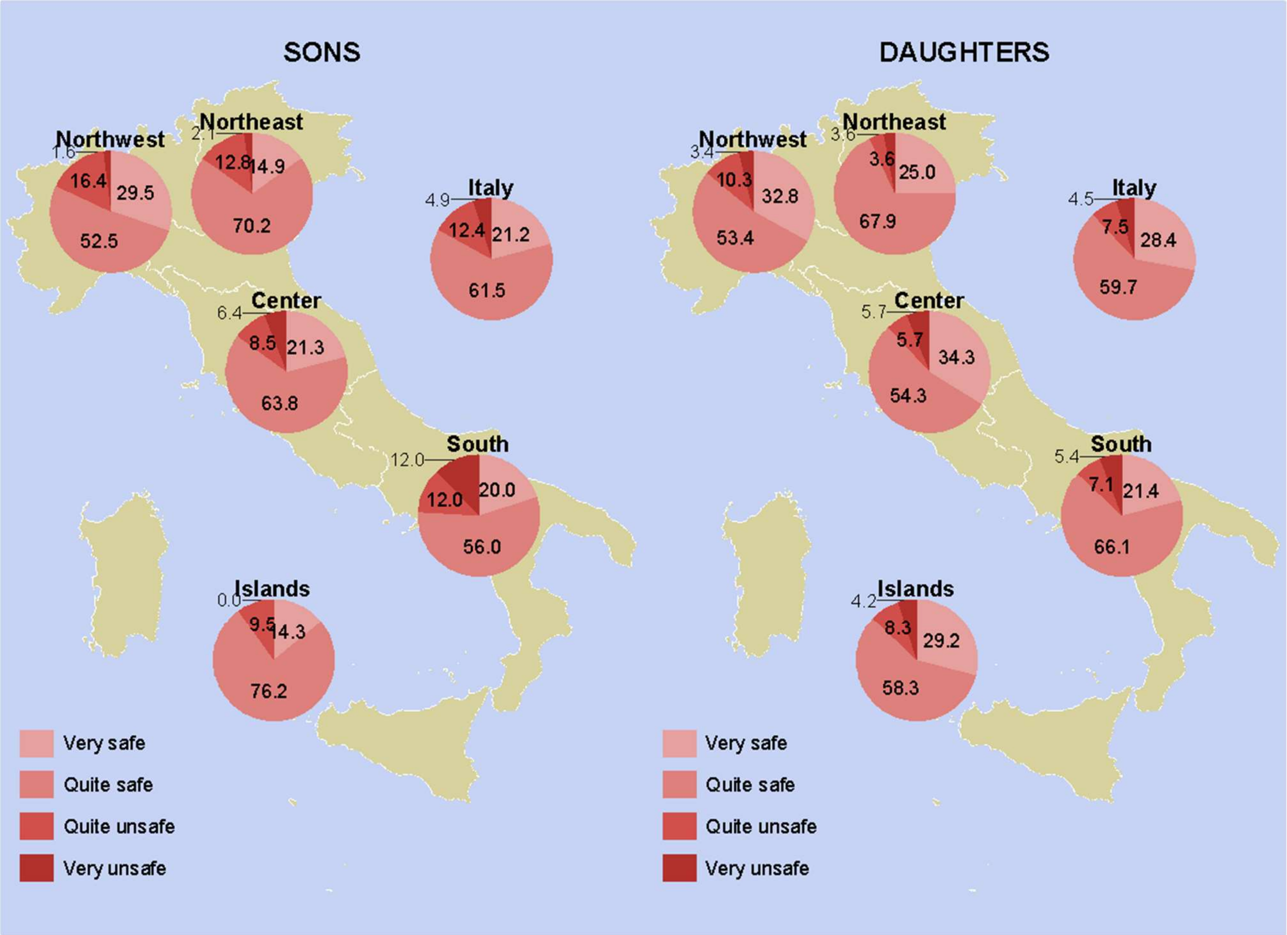

Notes: Information was provided by the parents of the children. Piedmont, Aosta Valley, Lombardy, and Liguria constitute Northwestern Italy; Trentino-South Tyrol, Veneto, Friuli-Venezia Giulia, and Emilia-Romagna constitute Northeastern Italy; Tuscany, Umbria, Marche, and Lazio constitute Central Italy; Abruzzo, Molise, Campania, Apulia, Basilicata, and Calabria constitute Southern Italy; Sicily and Sardinia constitute Insular Italy.
HPV, human papillomavirus; *NUTS*, Nomenclature of Territorial Units for Statistics.

Figure S8. Perception of the safety of HPV vaccine among male vs. female offspring between 12 and 17 years of age (*n* = 839), overall and by NUTS (%).

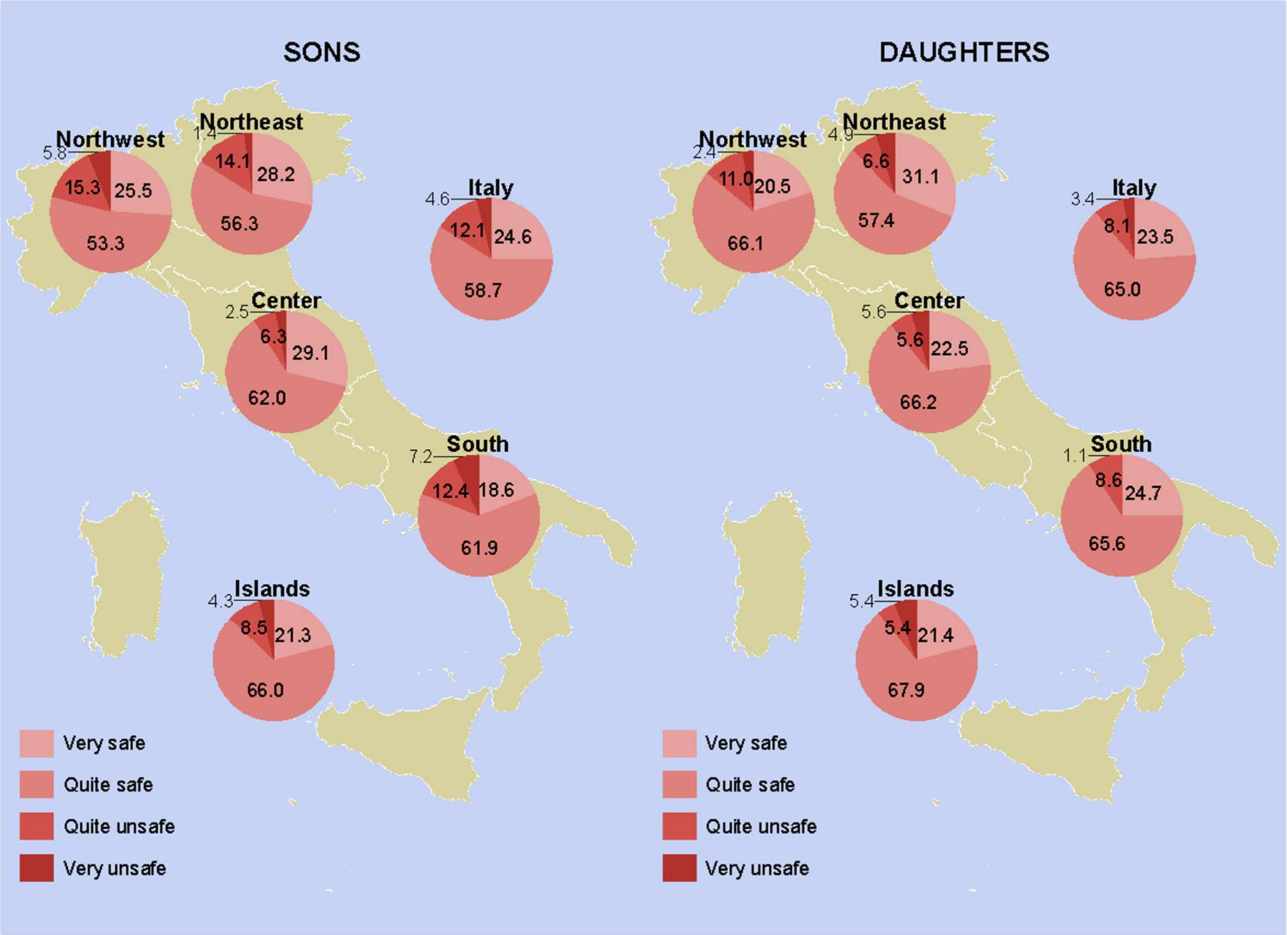

Notes: Information was provided by the parents of the children. Piedmont, Aosta Valley, Lombardy, and Liguria constitute Northwestern Italy; Trentino-South Tyrol, Veneto, Friuli-Venezia Giulia, and Emilia-Romagna constitute Northeastern Italy; Tuscany, Umbria, Marche, and Lazio constitute Central Italy; Abruzzo, Molise, Campania, Apulia, Basilicata, and Calabria constitute Southern Italy; Sicily and Sardinia constitute Insular Italy.
HPV, human papillomavirus; *NUTS*, Nomenclature of Territorial Units for Statistics.

Figure S9. Perception of how easy it is to access healthcare facilities to get an HPV vaccine among female participants 26 years of age and older who answered on their own behalf (n = 2109), overall and by NUTS.

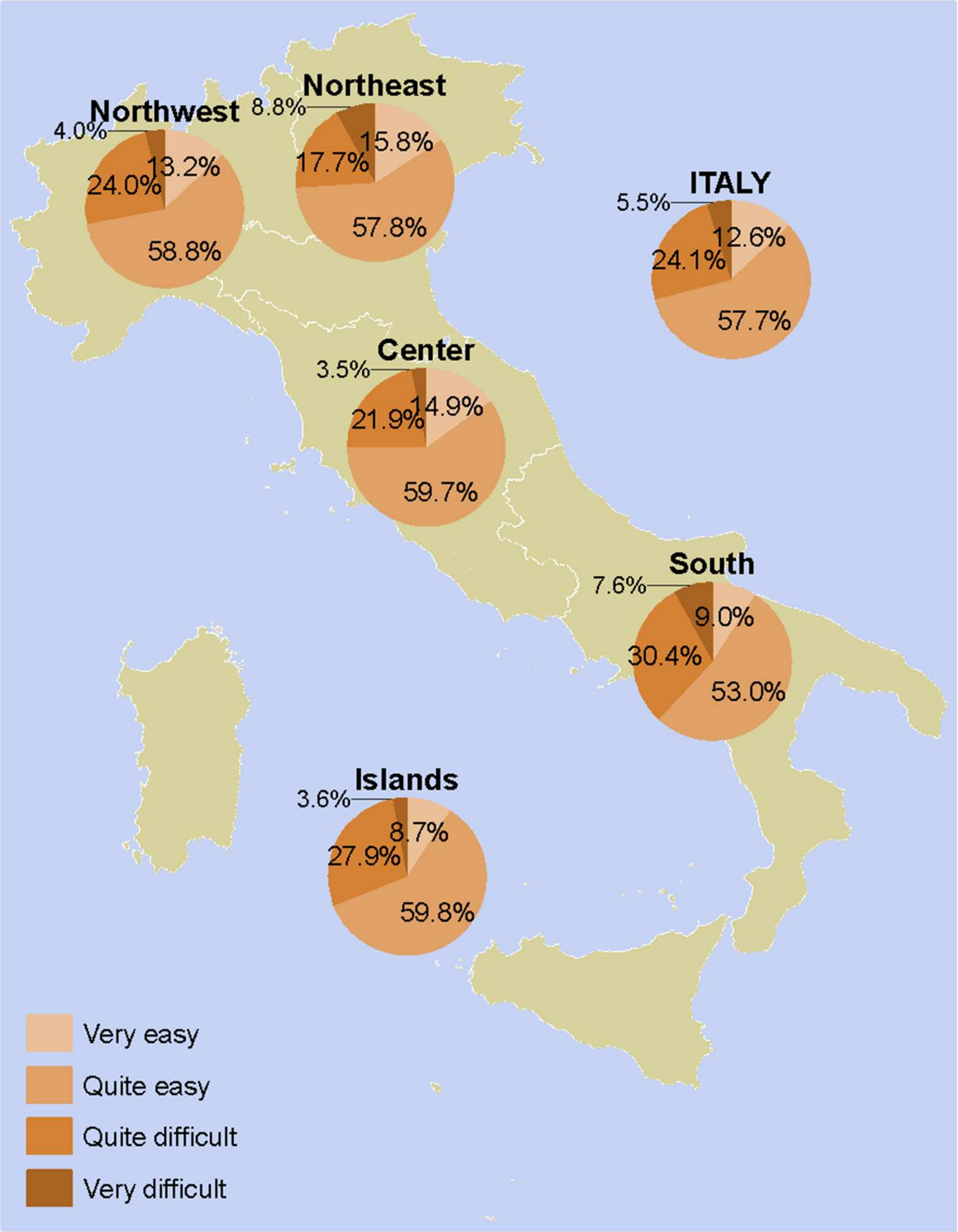

Notes: Females include non-binary people and participants who did not disclose their gender identity. Piedmont, Aosta Valley, Lombardy, and Liguria constitute Northwestern Italy; Trentino-South Tyrol, Veneto, Friuli-Venezia Giulia, and Emilia-Romagna constitute Northeastern Italy; Tuscany, Umbria, Marche, and Lazio constitute Central Italy; Abruzzo, Molise, Campania, Apulia, Basilicata, and Calabria constitute Southern Italy; Sicily and Sardinia constitute Insular Italy.
HPV, human papillomavirus; *NUTS*, Nomenclature of Territorial Units for Statistics.

Figure S10. Perception of how easy it is to access healthcare facilities to get an HPV vaccine among male vs. female participants between 18 and 25 years of age who answered on their own behalf (*n* = 1051), overall and by NUTS (%).

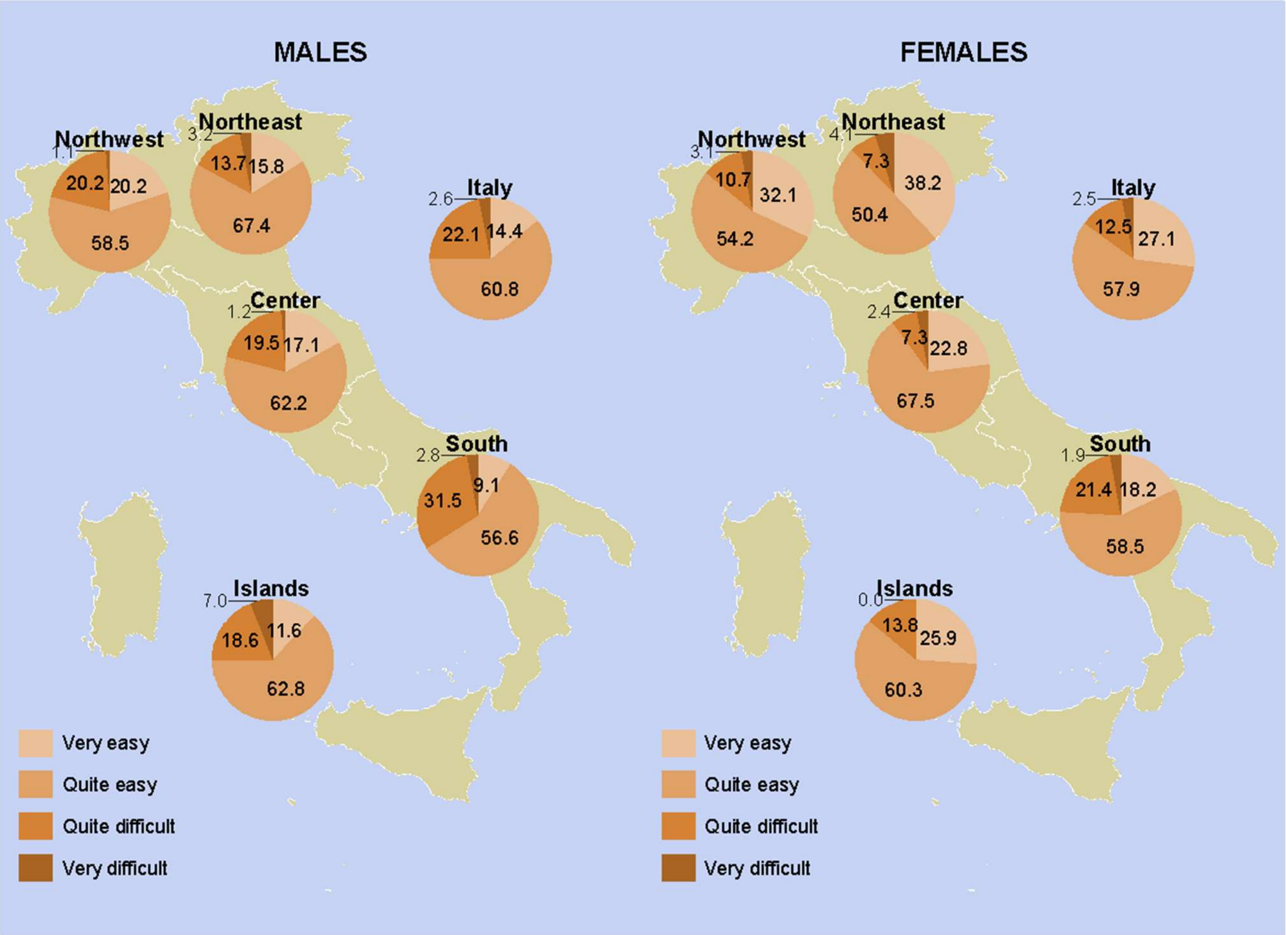

Notes: Females include non-binary people and participants who did not disclose their gender identity. Piedmont, Aosta Valley, Lombardy, and Liguria constitute Northwestern Italy; Trentino-South Tyrol, Veneto, Friuli-Venezia Giulia, and Emilia-Romagna constitute Northeastern Italy; Tuscany, Umbria, Marche, and Lazio constitute Central Italy; Abruzzo, Molise, Campania, Apulia, Basilicata, and Calabria constitute Southern Italy; Sicily and Sardinia constitute Insular Italy.
HPV, human papillomavirus; *NUTS*, Nomenclature of Territorial Units for Statistics.

Figure S11. Perception of how easy it is to access healthcare facilities to get an HPV vaccine among male vs. female offspring between 9 and 11 years of age (n = 427), overall and by NUTS (%).

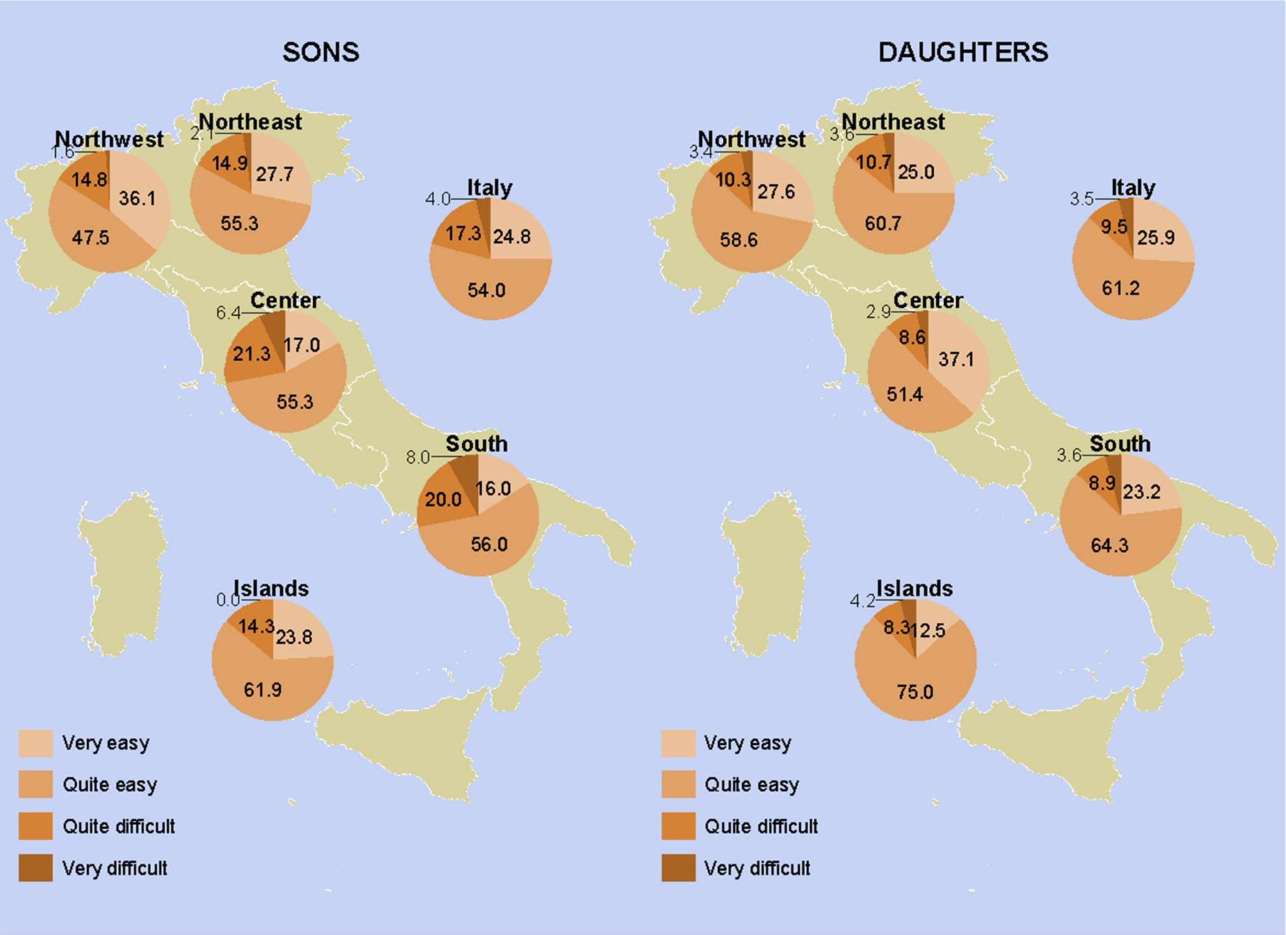

Notes: Information was provided by the parents of the children. Piedmont, Aosta Valley, Lombardy, and Liguria constitute Northwestern Italy; Trentino-South Tyrol, Veneto, Friuli-Venezia Giulia, and Emilia-Romagna constitute Northeastern Italy; Tuscany, Umbria, Marche, and Lazio constitute Central Italy; Abruzzo, Molise, Campania, Apulia, Basilicata, and Calabria constitute Southern Italy; Sicily and Sardinia constitute Insular Italy.
HPV, human papillomavirus; *NUTS*, Nomenclature of Territorial Units for Statistics.

Figure S12. Perception of how easy it is to access healthcare facilities to get an HPV vaccine among male vs. female offspring between 12 and 17 years of age (*n* = 839), overall and by NUTS (%).

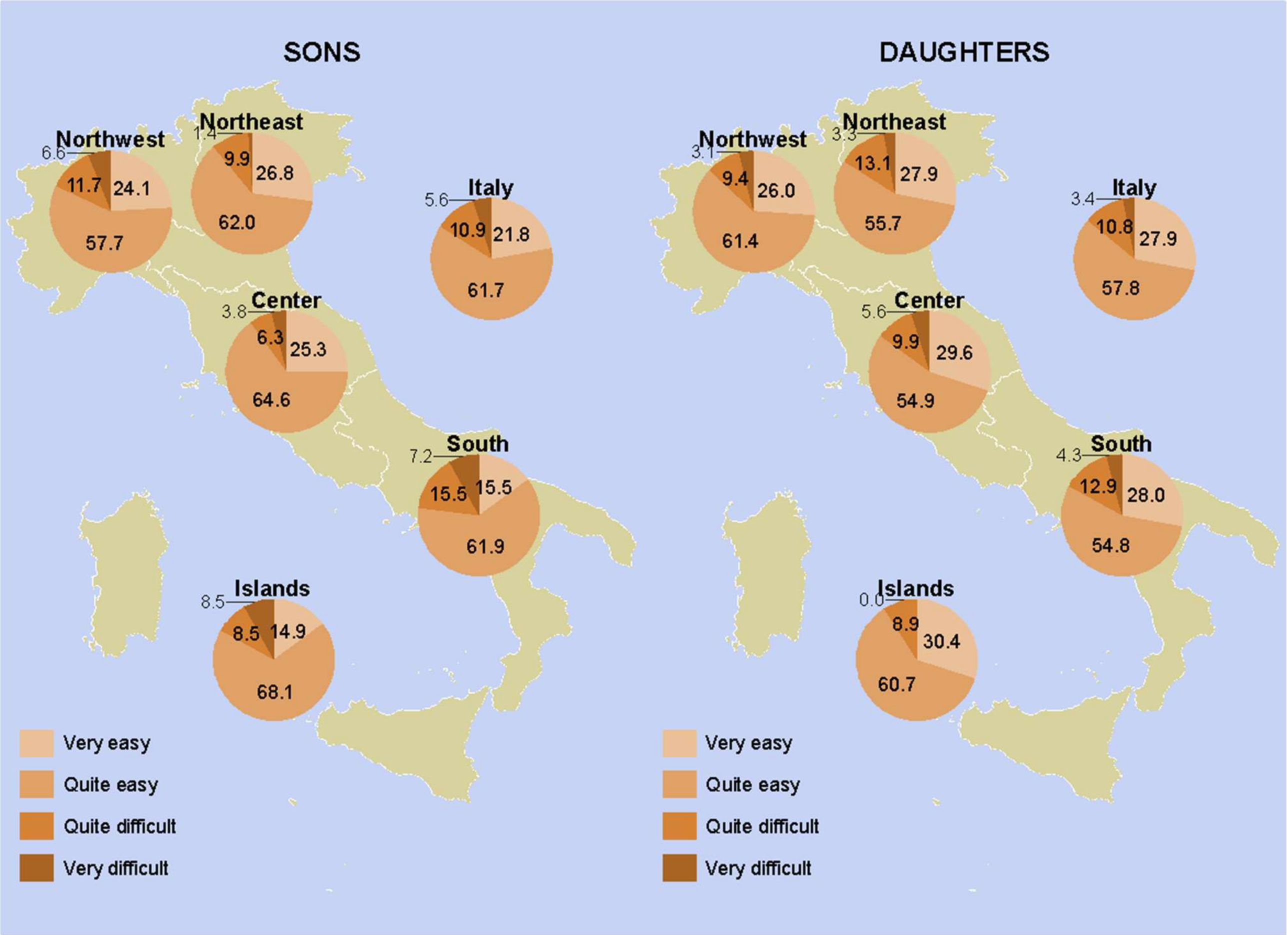

Notes: Information was provided by the parents of the children. Piedmont, Aosta Valley, Lombardy, and Liguria constitute Northwestern Italy; Trentino-South Tyrol, Veneto, Friuli-Venezia Giulia, and Emilia-Romagna constitute Northeastern Italy; Tuscany, Umbria, Marche, and Lazio constitute Central Italy; Abruzzo, Molise, Campania, Apulia, Basilicata, and Calabria constitute Southern Italy; Sicily and Sardinia constitute Insular Italy.
HPV, human papillomavirus; *NUTS*, Nomenclature of Territorial Units for Statistics.

Figure S13. Perception of the affordability of HPV vaccines among female participants 26 years of age and older who answered on their own behalf (n = 2109), overall and by NUTS.

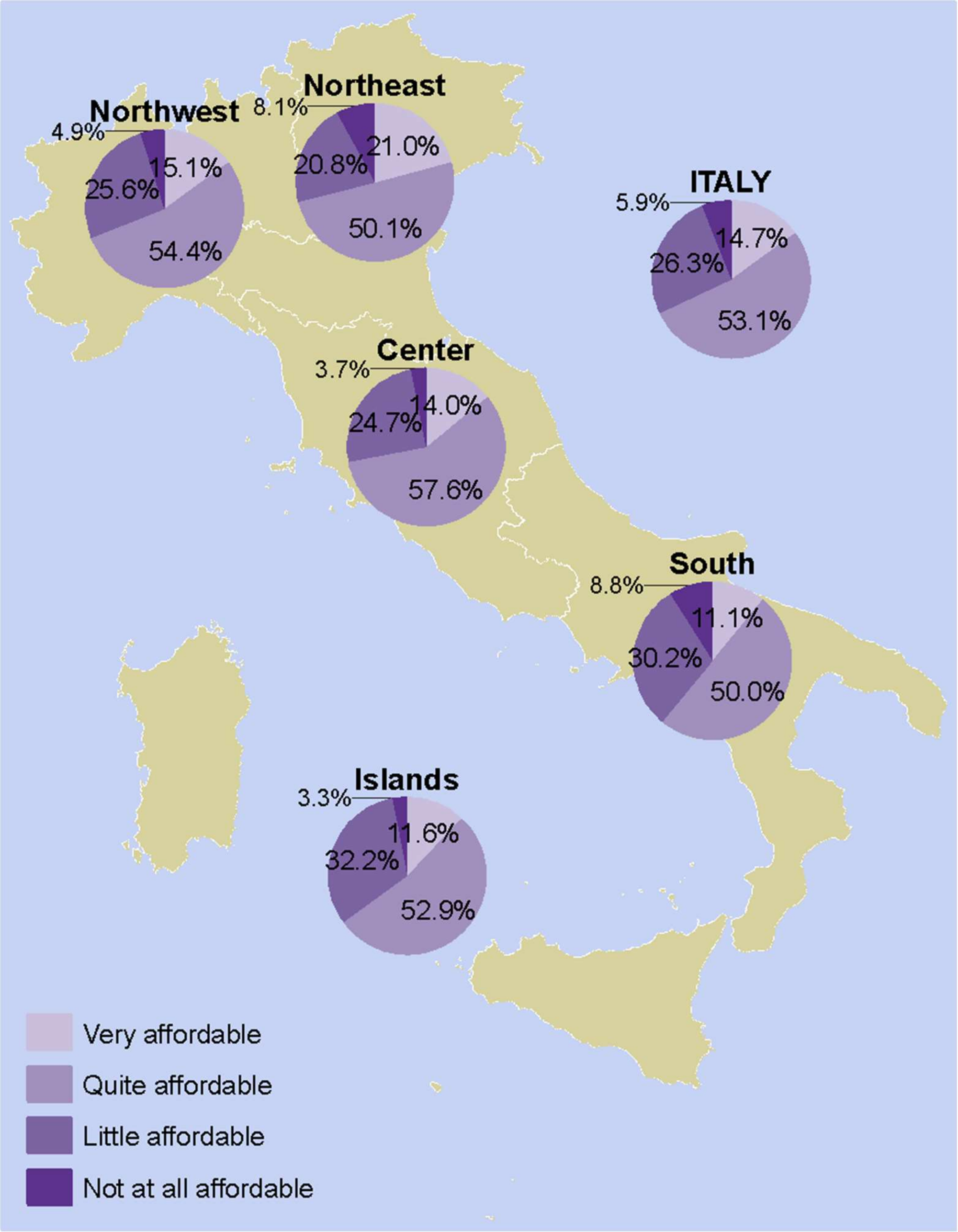

Notes: Females include non-binary people and participants who did not disclose their gender identity. Piedmont, Aosta Valley, Lombardy, and Liguria constitute Northwestern Italy; Trentino-South Tyrol, Veneto, Friuli-Venezia Giulia, and Emilia-Romagna constitute Northeastern Italy; Tuscany, Umbria, Marche, and Lazio constitute Central Italy; Abruzzo, Molise, Campania, Apulia, Basilicata, and Calabria constitute Southern Italy; Sicily and Sardinia constitute Insular Italy.
HPV, human papillomavirus; *NUTS*, Nomenclature of Territorial Units for Statistics.

Figure S14. Perception of the affordability of HPV vaccines among male vs. female participants between 18 and 25 years of age who answered on their own behalf (n = 1051), overall and by NUTS.

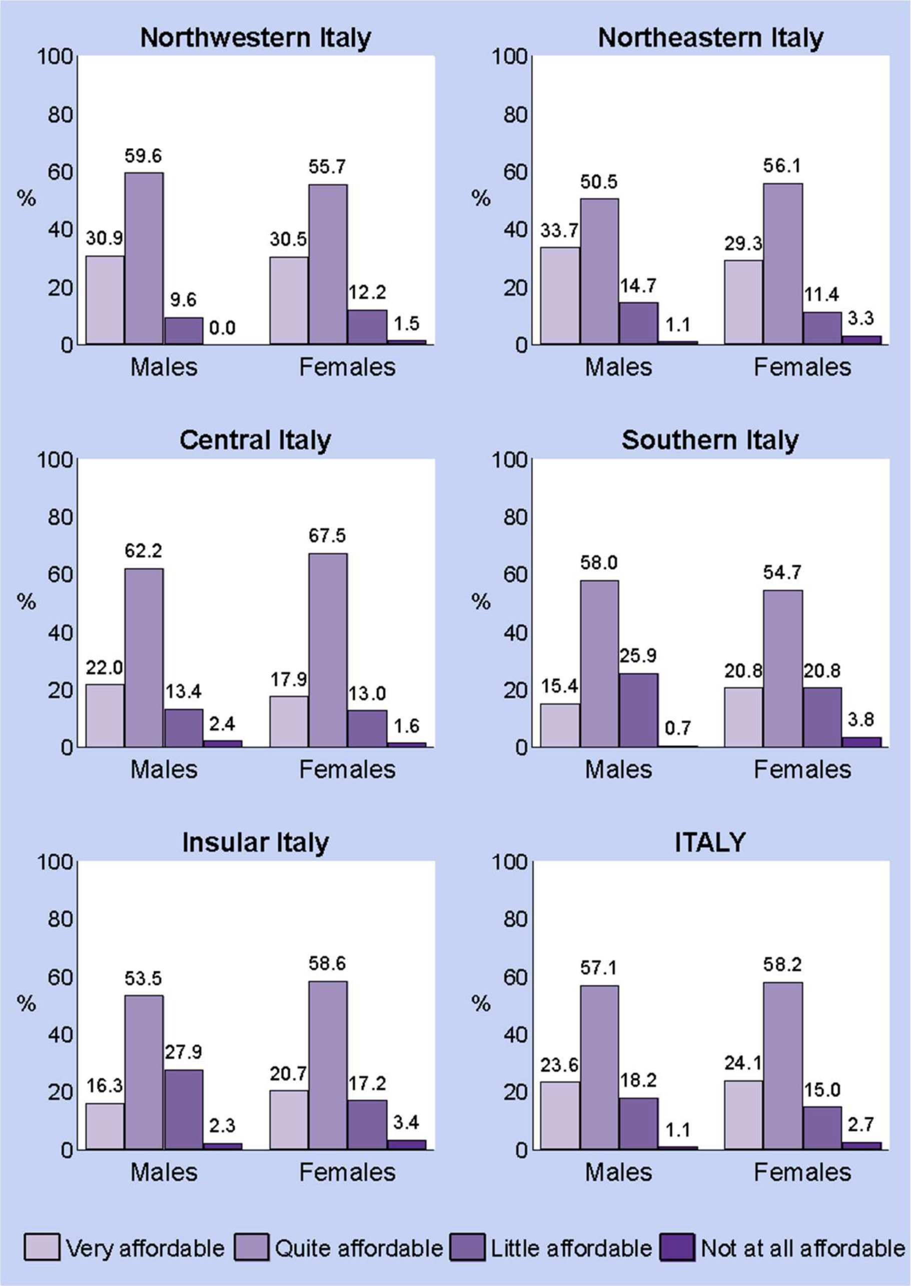

Notes: The presence of zero counts made it impossible to visualize the data as thematic maps using the 'spmap' Stata command. Females include non-binary people and participants who did not disclose their gender identity. Piedmont, Aosta Valley, Lombardy, and Liguria constitute Northwestern Italy; Trentino-South Tyrol, Veneto, Friuli-Venezia Giulia, and Emilia-Romagna constitute Northeastern Italy; Tuscany, Umbria, Marche, and Lazio constitute Central Italy; Abruzzo, Molise, Campania, Apulia, Basilicata, and Calabria constitute Southern Italy; Sicily and Sardinia constitute Insular Italy.
HPV, human papillomavirus; *NUTS*, Nomenclature of Territorial Units for Statistics.

Figure S15. Perception of the affordability of HPV vaccines among male vs. female offspring between 9 and 11 years of age (n = 427), overall and by NUTS.

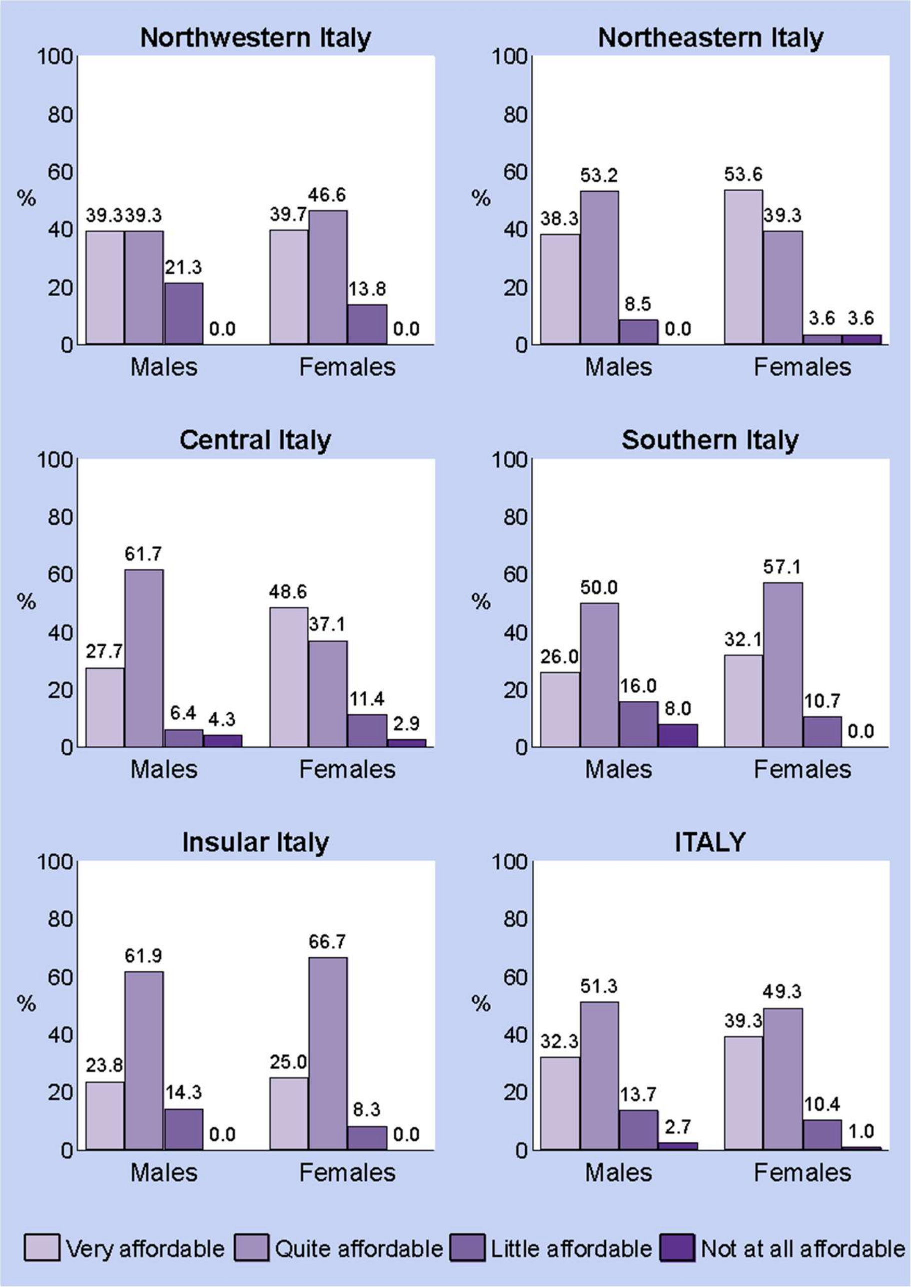

Notes: The presence of zero counts made it impossible to visualize the data as thematic maps using the 'spmap' Stata command. Information was provided by the parents of the children. Piedmont, Aosta Valley, Lombardy, and Liguria constitute Northwestern Italy; Trentino-South Tyrol, Veneto, Friuli-Venezia Giulia, and Emilia-Romagna constitute Northeastern Italy; Tuscany, Umbria, Marche, and Lazio constitute Central Italy; Abruzzo, Molise, Campania, Apulia, Basilicata, and Calabria constitute Southern Italy; Sicily and Sardinia constitute Insular Italy. *HPV*, human papillomavirus; *NUTS*, Nomenclature of Territorial Units for Statistics.

Figure S16. Perception of the affordability of HPV vaccines among male vs. female offspring between 12 and 17 years of age (n = 839), overall and by NUTS (%).

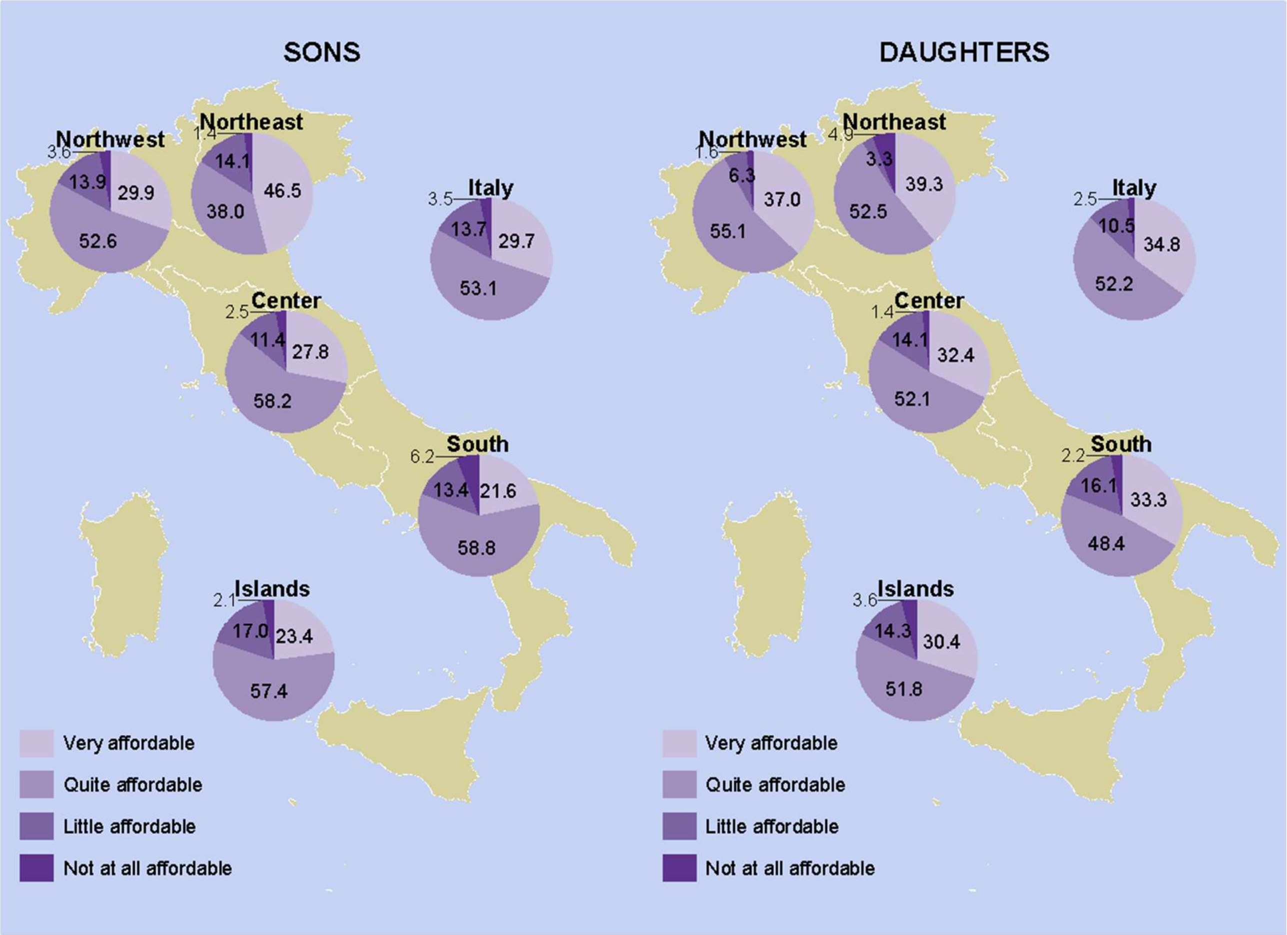

Notes: Information was provided by the parents of the children. Piedmont, Aosta Valley, Lombardy, and Liguria constitute Northwestern Italy; Trentino-South Tyrol, Veneto, Friuli-Venezia Giulia, and Emilia-Romagna constitute Northeastern Italy; Tuscany, Umbria, Marche, and Lazio constitute Central Italy; Abruzzo, Molise, Campania, Apulia, Basilicata, and Calabria constitute Southern Italy; Sicily and Sardinia constitute Insular Italy.
HPV, human papillomavirus; *NUTS*, Nomenclature of Territorial Units for Statistics.

Figure S17. Payment for HPV vaccination among female participants 26 years of age and older who answered on their own behalf, overall and by NUTS; only subjects vaccinated against HPV are included (*n* = 457).

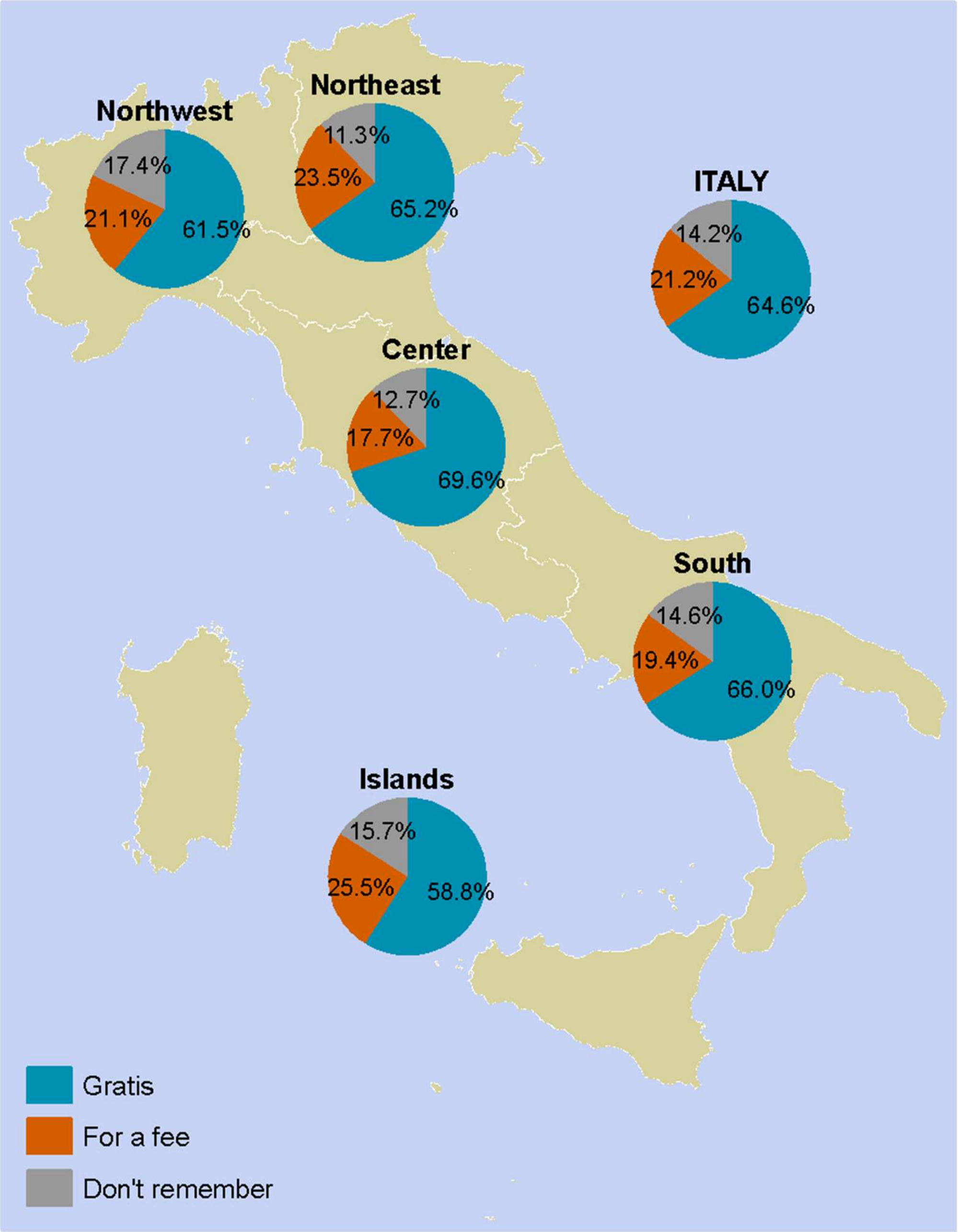

Notes: Females include non-binary people and participants who did not disclose their gender identity. Piedmont, Aosta Valley, Lombardy, and Liguria constitute Northwestern Italy; Trentino-South Tyrol, Veneto, Friuli-Venezia Giulia, and Emilia-Romagna constitute Northeastern Italy; Tuscany, Umbria, Marche, and Lazio constitute Central Italy; Abruzzo, Molise, Campania, Apulia, Basilicata, and Calabria constitute Southern Italy; Sicily and Sardinia constitute Insular Italy.
HPV, human papillomavirus; *NUTS*, Nomenclature of Territorial Units for Statistics.

Figure S18. Payment for HPV vaccination among male vs. female participants between 18 and 25 years of age, overall and by NUTS; only subjects vaccinated against HPV are included (n = 654).

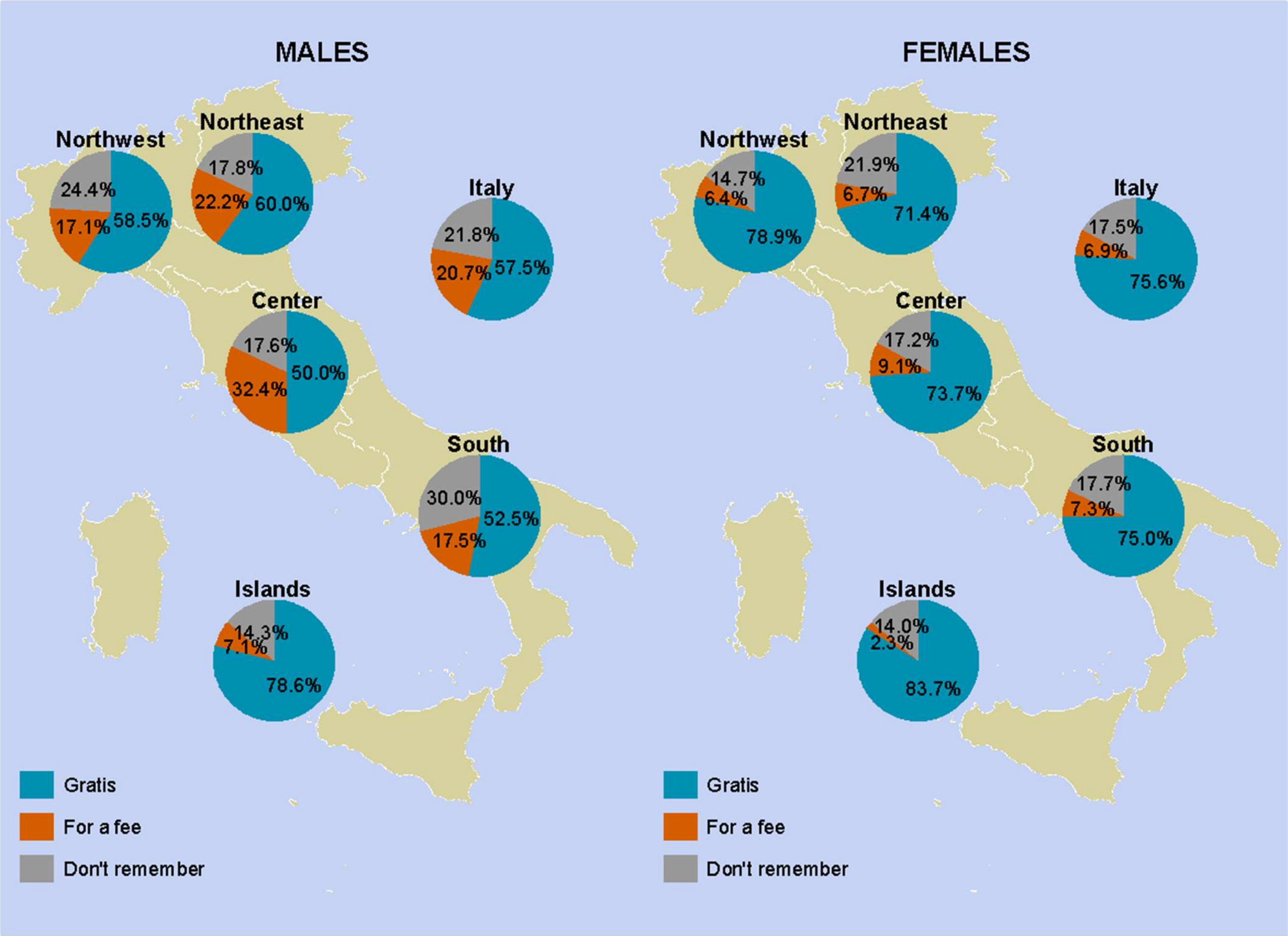

Notes: Females include non-binary people and participants who did not disclose their gender identity. Piedmont, Aosta Valley, Lombardy, and Liguria constitute Northwestern Italy; Trentino-South Tyrol, Veneto, Friuli-Venezia Giulia, and Emilia-Romagna constitute Northeastern Italy; Tuscany, Umbria, Marche, and Lazio constitute Central Italy; Abruzzo, Molise, Campania, Apulia, Basilicata, and Calabria constitute Southern Italy; Sicily and Sardinia constitute Insular Italy.
HPV, human papillomavirus; *NUTS*, Nomenclature of Territorial Units for Statistics.

Figure S19. Payment for HPV vaccination among male vs. female offspring between 9 and 11 years of age, overall and by NUTS; only subjects vaccinated against HPV are included (n = 171).

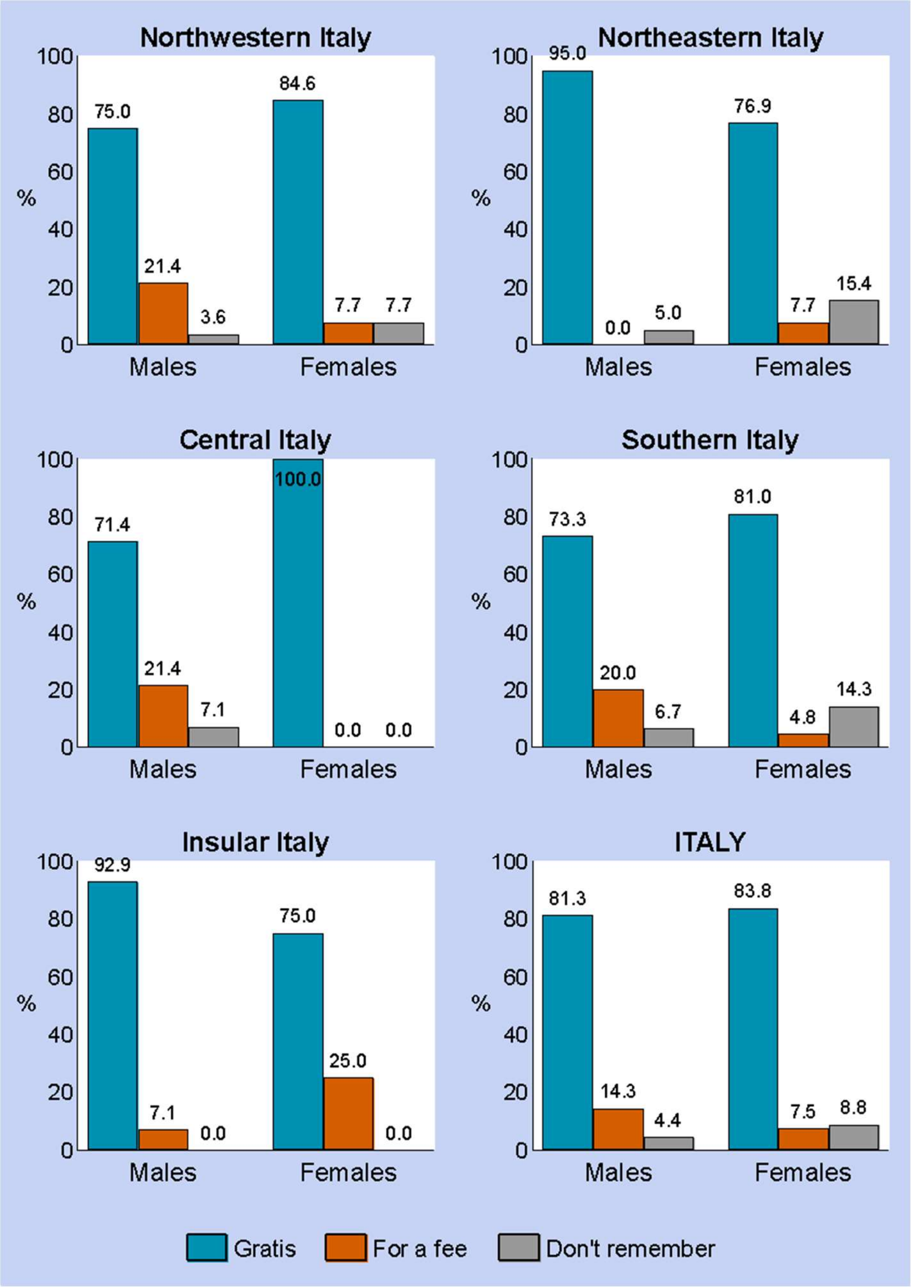

Notes: The presence of zero counts made it impossible to visualize the data as thematic maps using the 'spmap' Stata command. Information was provided by the parents of the children. Piedmont, Aosta Valley, Lombardy, and Liguria constitute Northwestern Italy; Trentino-South Tyrol, Veneto, Friuli-Venezia Giulia, and Emilia-Romagna constitute Northeastern Italy; Tuscany, Umbria, Marche, and Lazio constitute Central Italy; Abruzzo, Molise, Campania, Apulia, Basilicata, and Calabria constitute Southern Italy; Sicily and Sardinia constitute Insular Italy. *HPV*, human papillomavirus; *NUTS*, Nomenclature of Territorial Units for Statistics.

Figure S20. Payment for HPV vaccination among male vs. female offspring between 12 and 17 years of age, overall and by NUTS; only subjects vaccinated against HPV are included (n = 519).

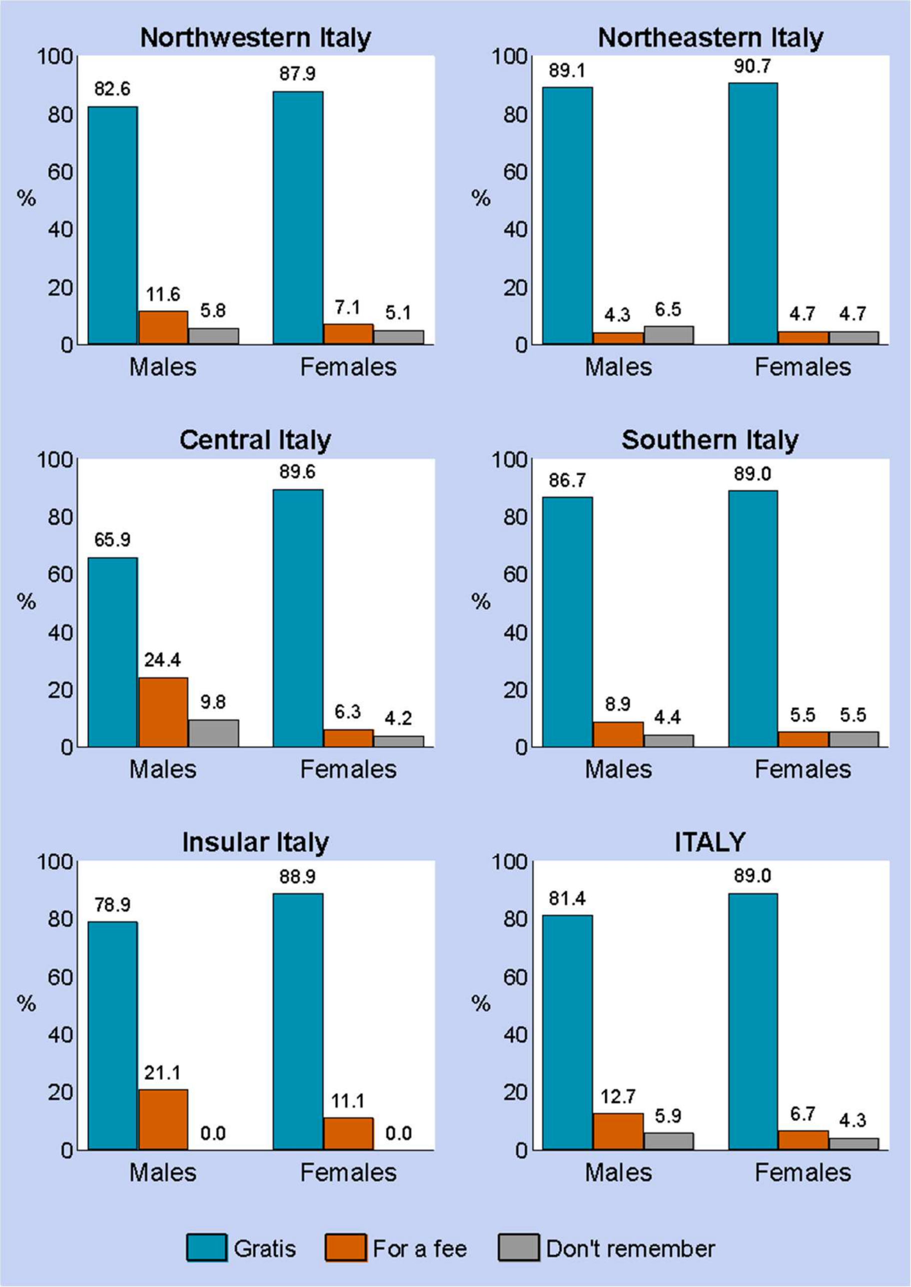

Notes: The presence of zero counts made it impossible to visualize the data as thematic maps using the 'spmap' Stata command. Information was provided by the parents of the children. Piedmont, Aosta Valley, Lombardy, and Liguria constitute Northwestern Italy; Trentino-South Tyrol, Veneto, Friuli-Venezia Giulia, and Emilia-Romagna constitute Northeastern Italy; Tuscany, Umbria, Marche, and Lazio constitute Central Italy; Abruzzo, Molise, Campania, Apulia, Basilicata, and Calabria constitute Southern Italy; Sicily and Sardinia constitute Insular Italy. *HPV*, human papillomavirus; *NUTS*, Nomenclature of Territorial Units for Statistics.

SUPPLEMENTARY MATERIAL – SURVEY TOOL

1. Sex

2. Year of birth

3. Education level

· Elementary/middle school

· High school

· University

· Postgraduate education

4. What is the postcode of the area you live in?

5. Occupation

· Student

· Doctor

· Other health worker

· Law enforcement

· Teacher

· Employed (other category than above)

· Unemployed

· Retired

6. Who do you live with?

· I live alone

· I live as a couple

· I live with my family of origin

· Other

7. With the financial resources available to you (from your own or your family's income) can you meet the needs of your current living situation?

· Grade 1 (with many difficulties)

· Grade 2

· Grade 3

· Grade 4

· Grade 5 (with no difficulties)

8. Do you have any children?

· Yes

· No

9. What is the sex of your youngest child?

· Male

· Female

10. Date of birth of the youngest child:

Free field: DD/MM/YYYY

11. Who makes decisions about vaccination of your children?

· Mainly me

· Mainly my partner

· Evenly divided

12. Were you pregnant at the beginning of the influenza epidemic season (October/November 2021)?

· Yes

· No

13. Due to a physical, psychological or sensory disability, do you have difficulties completing daily tasks such as going to the doctor or buying groceries?

· Yes

· No

14. Weight

Free range: kg

15. Height

Free range: cm

16. Do you have chronic respiratory diseases (i.e.: severe asthma, bronchopulmonary dysplasia, cystic fibrosis and chronic obstructive pulmonary disease-BPCO)?

· Yes

· No

17. Do you have any cardiovascular chronic diseases (i.e.: congenital and acquired heart disease)?

· Yes

· No

18. Are you diabetic?

· Yes

· No

19. In which of the following facilities did you have most of your vaccinations?

· In a hospital

· In a pharmacy

· At your family doctor's

· In a vaccination hub

· At home

· At work

20. If you could choose, in which facility/place would you prefer to receive a vaccination?

· In a hospital

· In a pharmacy

· At your family doctor’s

· In a vaccination hub

· At home

· At work

21. How do your family and friends feel about vaccinations?

· Grade 1 (strongly disagree)

· Grade 2

· Grade 3

· Grade 4

· Grade 5 (strongly agree)

22. Have you had the papillomavirus vaccine (papillomavirus causes cervical/vaginal cancer, anal cancer, condylomas and other cancers)?

· Yes

· No

· I don't know what it is

23. Would you get an HPV shot?

· Yes

· No

24. How worried are you about getting the HPV infection?

· Grade 1 (very little)

· Grade 2

· Grade 3

· Grade 4

· Grade 5 (very much)

25. How safe do you think the HPV vaccine is?

· Grade 1 (not very safe)

· Grade 2

· Grade 3

· Grade 4

· Grade 5 (very safe)

26. Do you think you are entitled to the HPV vaccine?

· Yes

· No

27. How easy do you think it is for you to access facilities to get the HPV shot?

· Grade 1 (with many difficulties)

· Grade 2

· Grade 3

· Grade 4

· Grade 5 (with no difficulties)

28. How affordable do you think HPV vaccination is?

· Grade 1 (not at all accessible)

· Grade 2

· Grade 3

· Grade 4

· Grade 5 (very accessible)

29. Were you given the HPV vaccine free of charge or did you pay to receive it?

· Free of charge

· Paid
